# Exploring the Reuse of Dredged Sediments as a Sustainable Alternative for Eco-Friendly Brick Manufacturing: A Comprehensive Review

**DOI:** 10.3390/ma19194190

**Published:** 2026-09-30

**Authors:** Amani Abdallah, Mohamed Lachheb, Feras Kafiah, Malek Alkasrawi, Zohir Younsi

**Affiliations:** 1 ICL, Junia, Université Catholique de Lille, LITL, F-59000 Lille, France; amani_abdallah22783@hotmail.com; 2 CRMN, Centre for Research on Microelectronics and Nanotechnology of Sousse, NANOMISENE Lab, LR16CRMN01, Technopark of Sousse, Sousse 4050, Tunisia; mohamed.lachheb@crmn.rnrt.tn; 3 School of Engineering Technology, Al Hussein Technical University, Amman 11831, Jordan; feras.kafiah@htu.edu.jo; 4 Univ. Artois, IMT Nord Europe, Junia, Univ. Lille, ULR 4515, Laboratoire de Génie Civil et géo-Environnement (LGCgE), F-62400 Béthune, France; zohir.younsi@junia.com

**Keywords:** dredged sediments, brick production, sustainability, multi-criteria analysis, alternative material

## Abstract

The rapid growth of construction has significantly raised the hazard of the depletion of natural raw materials and posed a serious problem to the ecosystem’s stability. To establish an efficient policy for sustainable development, it is crucial to encourage responsible production practices, notably the promotion of waste recycling in construction materials production. In this context, reusing dredged sediments has emerged as a promising alternative to non-renewable resources in construction material manufacturing. This paper reviews recent research on valorizing dredged sediment as an alternative raw material in brick and block production. Furthermore, the study examines the mechanical, thermal, and environmental performance of bricks and blocks incorporating dredged sediments. Additionally, future research guidance is proposed to address existing challenges and to support the sustainable valorization of dredged materials within the construction sector.

## 1. Introduction

### 1.1. Background

Over the last few decades, global urbanization has grown exponentially, aligning with the increase in the world population. Consequently, this surge has led to a rising demand for construction materials, with global consumption reaching approximately 45 billion tons annually [1]. The use of construction materials is growing steadily. This presents the building industry with a growing challenge of long-term depletion of raw materials. Similarly, about 36% of the world’s energy consumption and 39% of the world’s CO_2_ emissions are due to the buildings’ construction and operation phases [2].

To address these challenges, researchers are working hard to develop green and sustainable construction materials that contribute to the conservation of natural resources. A particularly promising strategy involves the recycling and reuse of waste-derived alternative materials in construction manufacturing. Many researchers have investigated the incorporation of various types of waste, such as industrial [3,4,5], agricultural [6,7], and other by-products in construction materials [8,9]. These investigations have assessed the effects of incorporating these wastes into the material’s mechanical and thermal performances, along with other benefits such as environmental protection and energy-saving effects [10]. The above approach has several advantages. It preserves non-renewable natural resources, transforms waste into resources, and reduces greenhouse gas emissions [11]. Furthermore, these practices are fully aligned with European and French policy on waste management and sustainable construction, contributing to the shift towards a circular economy.

Among the sustainable alternative materials that can be repurposed in the construction industry, dredged sediments traditionally considered as waste are being increasingly considered due to the large volumes generated annually [12,13,14]. Sediments are waste materials excavated from the bottom of water bodies such as rivers, lakes, and harbors to restore the depth required for navigation and to maintain the ecological balance of the water body. They are inexpensive, and their abundance ensures affordability. Each year, 600 million cubic meters of sediment are dredged in ports and waterways to ensure safe navigation [15]. Europe alone contributes nearly half of this total of approximately 300 million cubic meters [16], with France contributing about 50 million cubic meters [17]. China engages in important dredging, approximately 100 million cubic meters, and the United States, where the number reaches 300 million cubic meters each year [18]. However, the management of this large volume of sediments generated through dredging presents significant ecological and environmental challenges [19]. As a result, there is an urgent need for innovative and sustainable solutions to transform these dredged sediments into valuable resources.

For the last two decades, research has been conducted worldwide for the development of innovative solutions and methodologies for the valorization of sediments [20,21,22]. Recent studies suggest dredged sediments can be used as raw materials for several civil engineering applications, including road construction [23,24,25,26], and also for the production of other construction materials like bricks [27], paving blocks [28], composite materials [29,30] and cementitious materials [31,32,33]. Various formulations of dredged sediments, often treated with binders such as cement [15,26] and lime [34] to enhance their mechanical properties, have been successfully integrated into different road layers, meeting design standards. The dredged sediment can either be the primary raw material for brick and block production or a partial substitute for sand [35,36,37]. The dredged sediment may also be used as a partial replacement for regular raw materials for Portland cement.

Over the past decade, numerous review papers have been published, which focus on the recycling and valorization opportunities of dredged sediments [38]. These reviews have extensively examined the beneficial uses and management processes involved in sediment reuse. Other reviews have examined the environmental impacts of dredging, dredging technologies, and sediment characteristics [39]. Furthermore, some studies particularly highlight the transformation of dredged sediment through multiple techniques to upcycle it into building materials, including aggregate, brick, conventional binder replacement, and alkaline activation [40]. Moreover, several papers have focused specifically on incorporating dredged sediments into cementitious materials [12,41,42]. Although there is growing interest in sediment reutilization, few review studies have systematically looked at how dredged sediments affect the thermal, mechanical and environmental performance of bricks and blocks, which are widely used in the construction sector. In this context, this paper focuses on exploring the incorporation of dredged sediments in brick and block manufacturing.

### 1.2. Analysis of Published Papers

Many previous works have studied the valorization of dredged sediments for various applications. In particular, the construction sector has emerged as a promising field for sediment reuse. Studies have shown that dredged sediments can be effectively utilized as raw constituents in construction materials such as concrete, mortar, and road construction materials.

**Figure 1:**
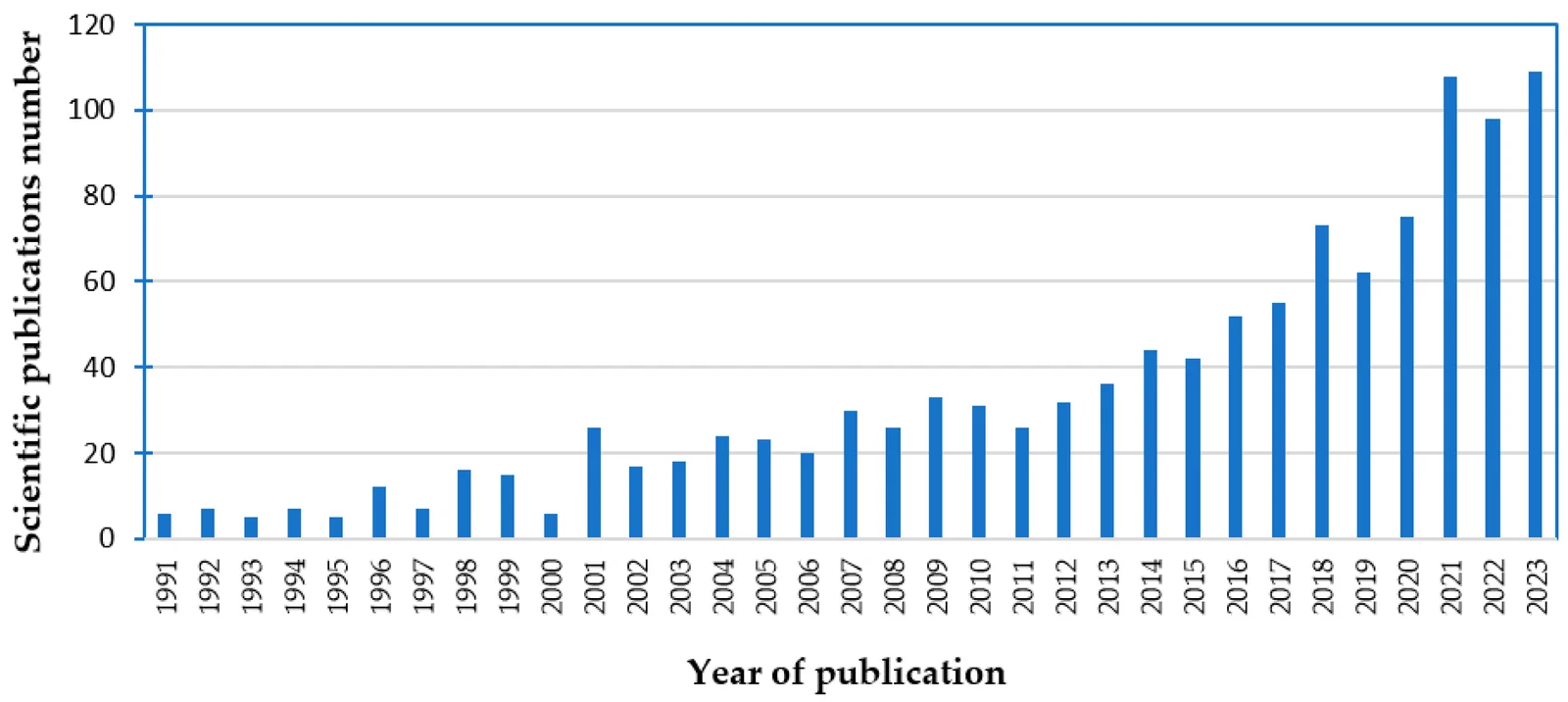
The scientific publications produced between 1991 and 2024 regarding the valorization of dredged sediments in construction materials.

Figure 1 shows the increasing academic interest in this field, based on data from the “Web of Science” core collection from 1991 to 2024. A structured bibliographic search was conducted on dredged sediments, reuse of materials (which includes recycling and valorization), and construction materials. The study considered only peer-reviewed journal articles and review papers. Results were then filtered and analyzed to ensure relevance to the technical performance, ecological impact and sustainability of sediment reuse in construction. Refinement was performed by selecting papers related to engineering, environmental science, and materials science.

This interest shows the ability of sediments to partially or fully replace the natural sand and aggregates used for preparing eco-friendly construction materials. It helps accelerate innovative practices and sustainable alternatives aligned with the circular economy, enhanced through cooperative research and engagement with industry in this field.

**Figure 2:**
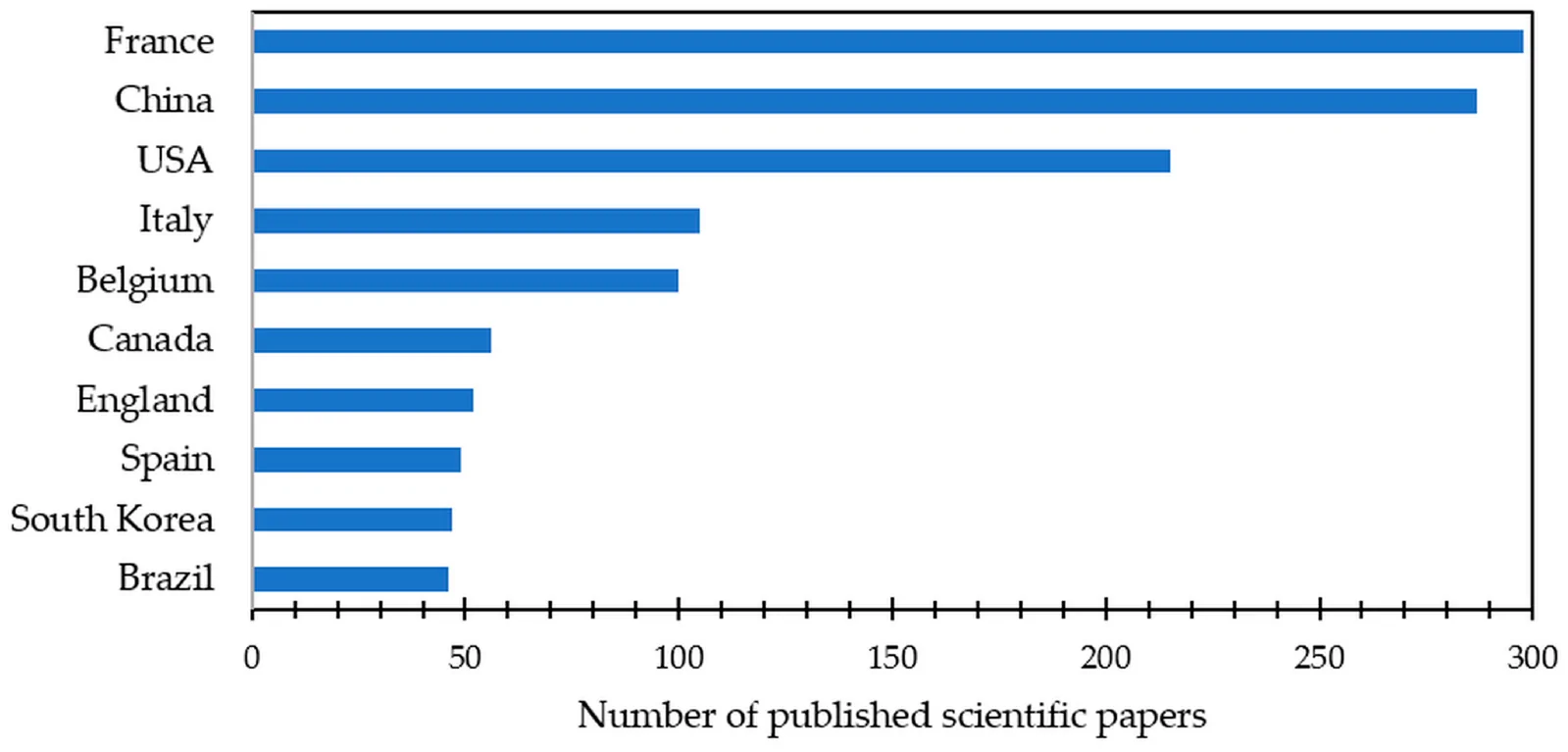
The countries with the maximum number of published papers on dredged sediments from 1991 to 2024.

As illustrated in Figure 2, the top 10 countries are presented with respect to the published papers from the earlier structured bibliographic search. France is the leading country in research on the valorization of dredged sediments, with 298 publications, ahead of major countries like China (287) and the USA (215). This highlights France’s strong commitment to sustainable sediment management, driven by both strict regulations and the need to reduce the environmental footprint from the construction sector.

The high research output of the top 10 countries reflects an active integration of circular economy principles into civil engineering practices, particularly through the reuse of dredged materials in construction applications. This also indicates strong collaboration between academia, industry, and public institutions to transform sediment waste into valuable resources while addressing different challenges.

**Figure 3:**
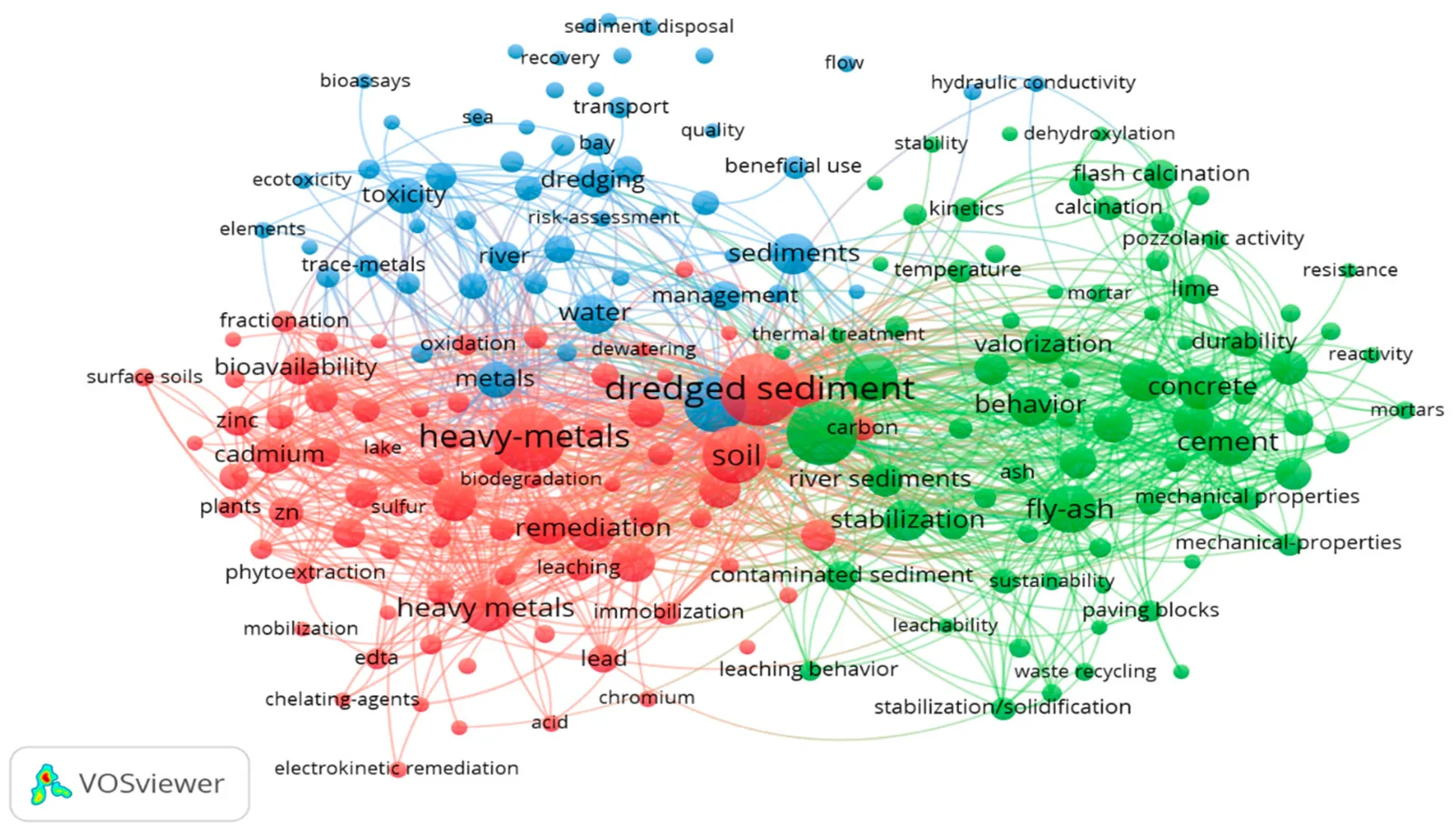
Keyword co-occurrence network visualization using VOSviewer. 3 Clusters, 6519 links, 15,134 total link strength.

Figure 3 presents a keyword co-occurrence network visualization constructed using VOSviewer (v.1.6.20), based on the bibliographic data collected. A total of 4445 keywords were detected, of which only those that had eight occurrences were selected. The network consists of three clusters, which are colored to show the most important themes in the literature. The map consists of 209 nodes and 6519 links, with a total link strength of 15,134, indicating a multidisciplinary research field.

The red cluster, containing 78 words, primarily focuses on contaminant-related research. Specifically, keywords on heavy metals, immobilization, and remediation were used. This cluster refers to a perspective in environmental science that highlights sediment pollution characterization and remediation techniques. The green cluster (77 words) focuses on valorization and construction applications, including concrete, cement, fly ash, durability, mechanical properties and stabilization. The cluster aligns with civil engineering and materials science research on transforming dredged sediments into construction materials. The blue cluster refers to the group of sediment management, which is about the disposal, transport and assessment of sediment. Studies within this cluster evaluated the origin, behavior and management of dredged material in aquatic environments.

**Figure 4:**
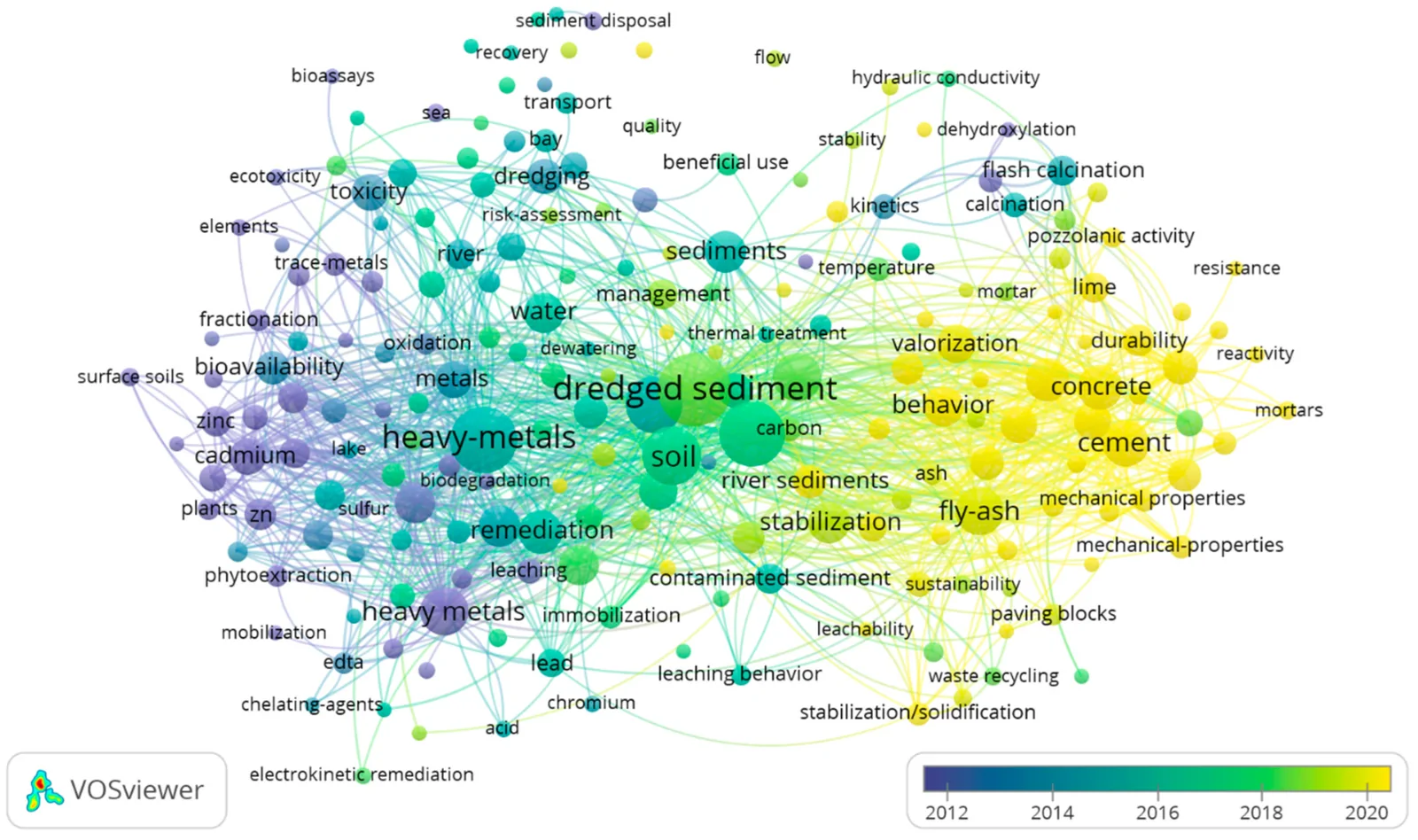
Overlay visualization of keyword co-occurrence by average publication year (2012–2020), generated using VOSviewer.

Figure 4 presents an overlay visualization of keyword co-occurrence, illustrating the temporal evolution of research trends related to dredged sediment reuse. The color gradient from dark blue (older terms, around 2012) to bright yellow (newer terms, around 2020) represents the average publication year for the keywords. Older research efforts (dark blue to green), particularly from 2012 to 2015, focused on contamination issues, remediation techniques, and metal toxicity, as evidenced by terms such as heavy metals, cadmium, bioavailability, and phytoextraction. This indicates an early emphasis on environmental risk and sediment quality assessments. More recent studies (green to yellow, ~2017 to 2020) have shifted towards valorization and civil engineering application, with keywords like cement, concrete, mechanical properties, durability, fly-ash and stabilization/solidification appearing in yellow. This reflects a growing interest in sustainable construction materials and the integration of treated sediments into material formulation strategies.

It should be noted that the bibliometric analysis was conducted to encompass the entire research field of dredged sediment valorization in construction and environmental applications in order to identify the main scientific trends, research interests, and thematic evolution. Therefore, the resulting keyword networks include topics related to cement, concrete, remediation, transportation, and waste management. In contrast, the subsequent sections of this review focus on the valorization of dredged sediments for brick production, which constitutes the primary objective and scope of the present work.

AI-assisted tools were used to support the graphical preparation and modification of selected figures. All scientific data, numerical values, figure content, interpretations, and conclusions were derived from the cited literature and verified by the authors. AI-assisted tools were not used to generate or modify scientific data or results.

### 1.3. Focus and Research Questions

In response to the construction industry’s resource depletion challenge, this review paper investigates the latest research on valorizing dredged sediments as secondary raw materials in diverse civil engineering applications. We further explore their potential to create new, sustainable construction materials.

Our aim is to facilitate decision-making for researchers and builders by providing insights into the optimal approaches for incorporating sediments in construction material manufacturing and assessing their implications for mechanical, durability, and environmental performance.

To achieve this goal, this review first introduces the origins, composition, and management challenges of dredged sediments. It then examines sediment valorization in brick and block production and critically evaluates physical, mechanical, thermal, durability, environmental, and economic performance. The final sections discuss implementation challenges, strategic recommendations, and future research needs.

## 2. Overview of the Dredged Sediments

### 2.1. Definition and Origin of Sediments

Sediments are generally defined as loose particles composed of sand, silt, and clay transported by natural processes [43]. They are essential for the ecological and geomorphological functioning of river basins, coastal zones, and estuarine environments. Sediments originate from rock and soil particles that are transported through riverine, coastal, and marine processes.

**Figure 5:**
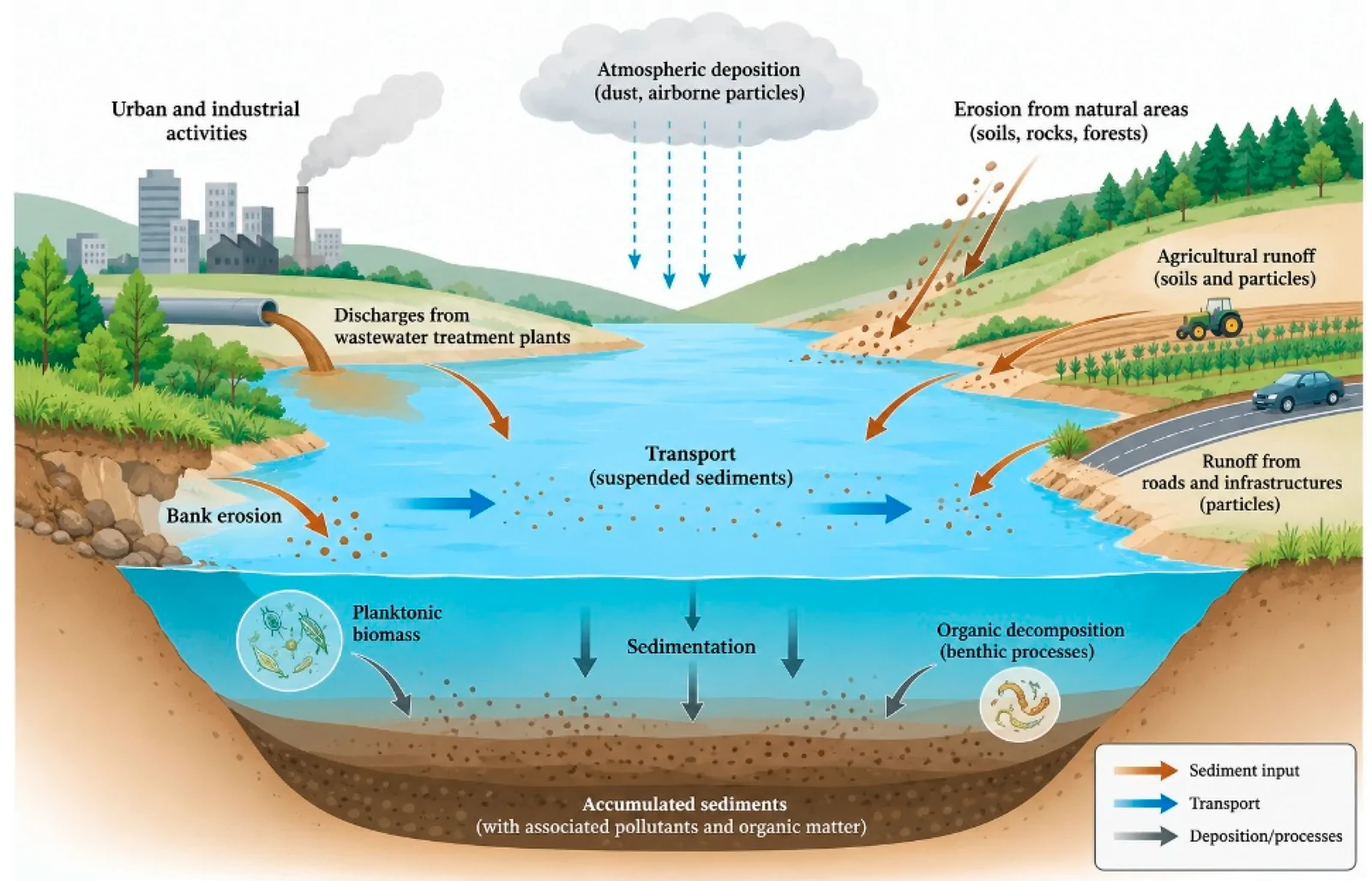
Schematic representation of the principal natural and anthropogenic sources, transport pathways, and accumulation processes of sediments in aquatic environments, reconstructed from [44].

Sediment transport in waterways involves several processes, including saltation, suspension, and rolling. The distribution of sediment particle sizes is largely influenced by water flow and transport dynamics, as illustrated in Figure 5.

Sediments differ in their characteristics depending on their composition, morphology, and origin. Sediment origins can either be endogenous or exogenous according to Schneider’s classification [45]. Endogenous sediments have their origin in the environment. For example, they may be formed through the decay of aquatic organisms. This includes organisms such as fauna or plant debris. In contrast, exogenous sediments are sourced from farther away and are transported by fluids (water, air, etc.). Table 1 presents an overview of the natural formation and sedimentation processes associated with sediments.

### 2.2. Composition of Sediments

**Figure 6:**
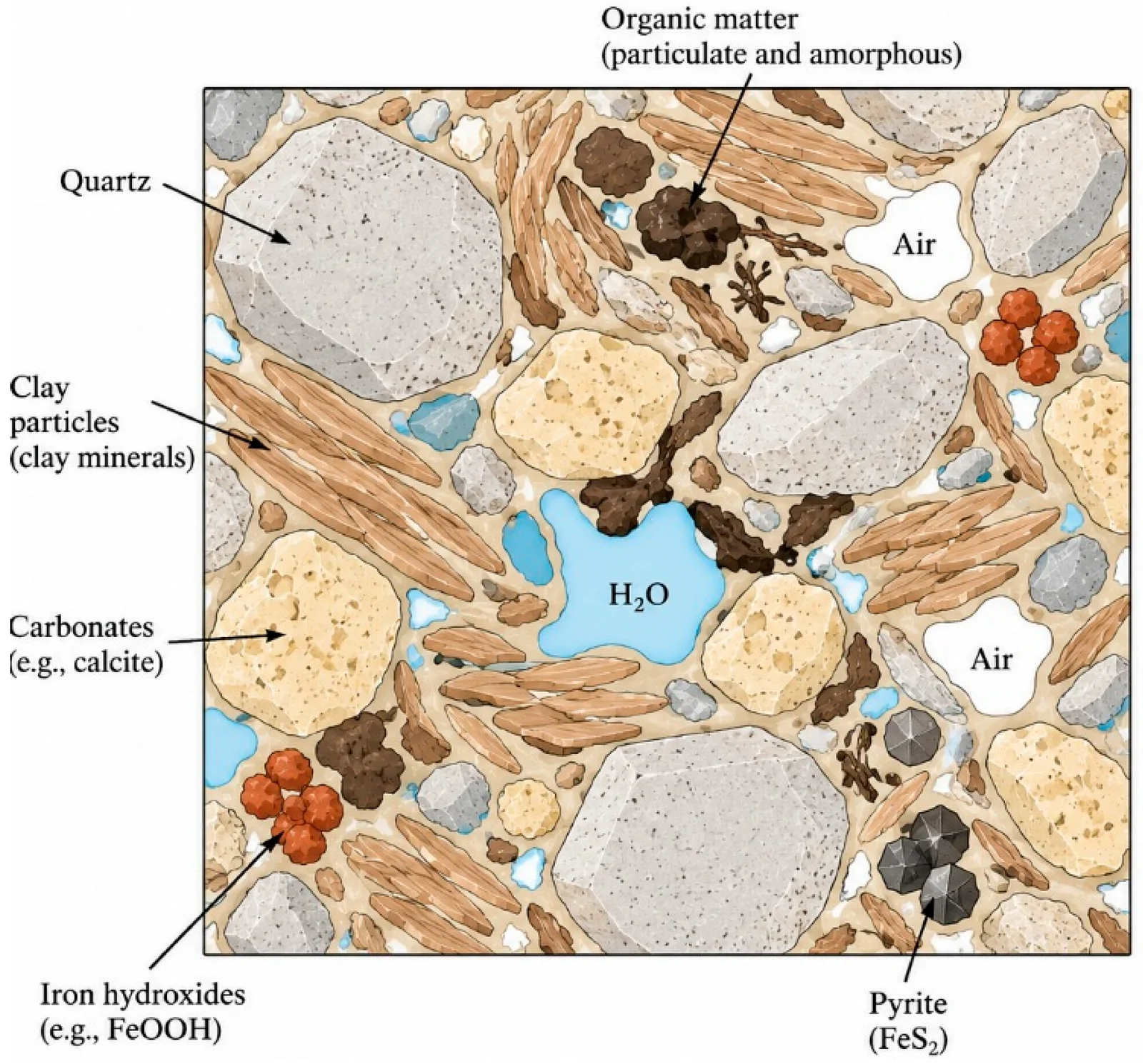
Schematic representation of the principal mineral, organic, and pore constituents of dredged sediments, reconstructed from [55].

Sediment composition is complex and highly variable. It is primarily defined by several key parameters, including granulometry (particle size distribution), mineralogical composition, water content, organic matter, and the presence of trace elements such as heavy metals, organic pollutants, and micro-pollutants [54]. The physiochemical properties of the sediments are governed by their constituents, which determine their behavior, transport and ecological impact within aquatic environments. The detailed composition and associated elements of sediments are illustrated in Figure 6.

To provide a comprehensive overview of the compositional characteristics of dredged sediments, Table 2 compares the major oxide compositions and dominant mineralogical phases of representative sediments from different geographical regions with those of conventional geomaterials, including ceramic clay, clayey soil, and quartz sand. This comparison highlights the considerable physicochemical variability of dredged sediments while illustrating their similarities with conventional geomaterials commonly used in construction materials.

As shown in Table 2, silica (SiO_2_) is the predominant oxide in both conventional geomaterials and most dredged sediments, followed by alumina (Al_2_O_3_), whereas calcium oxide (CaO) exhibits the greatest variability depending on sediment origin. Likewise, quartz is the dominant crystalline phase in nearly all sediments, while illite, kaolinite, montmorillonite, feldspars, and calcite are frequently identified according to the depositional environment. These observations demonstrate that many dredged sediments exhibit chemical and mineralogical characteristics comparable to those of conventional geomaterials, although significant compositional variability exists among different sediment sources. Such variability, particularly in the contents of CaO, alkali oxides, and loss on ignition (LOI), reflects differences in geological origin, depositional conditions, and organic matter content.

**Figure 7:**
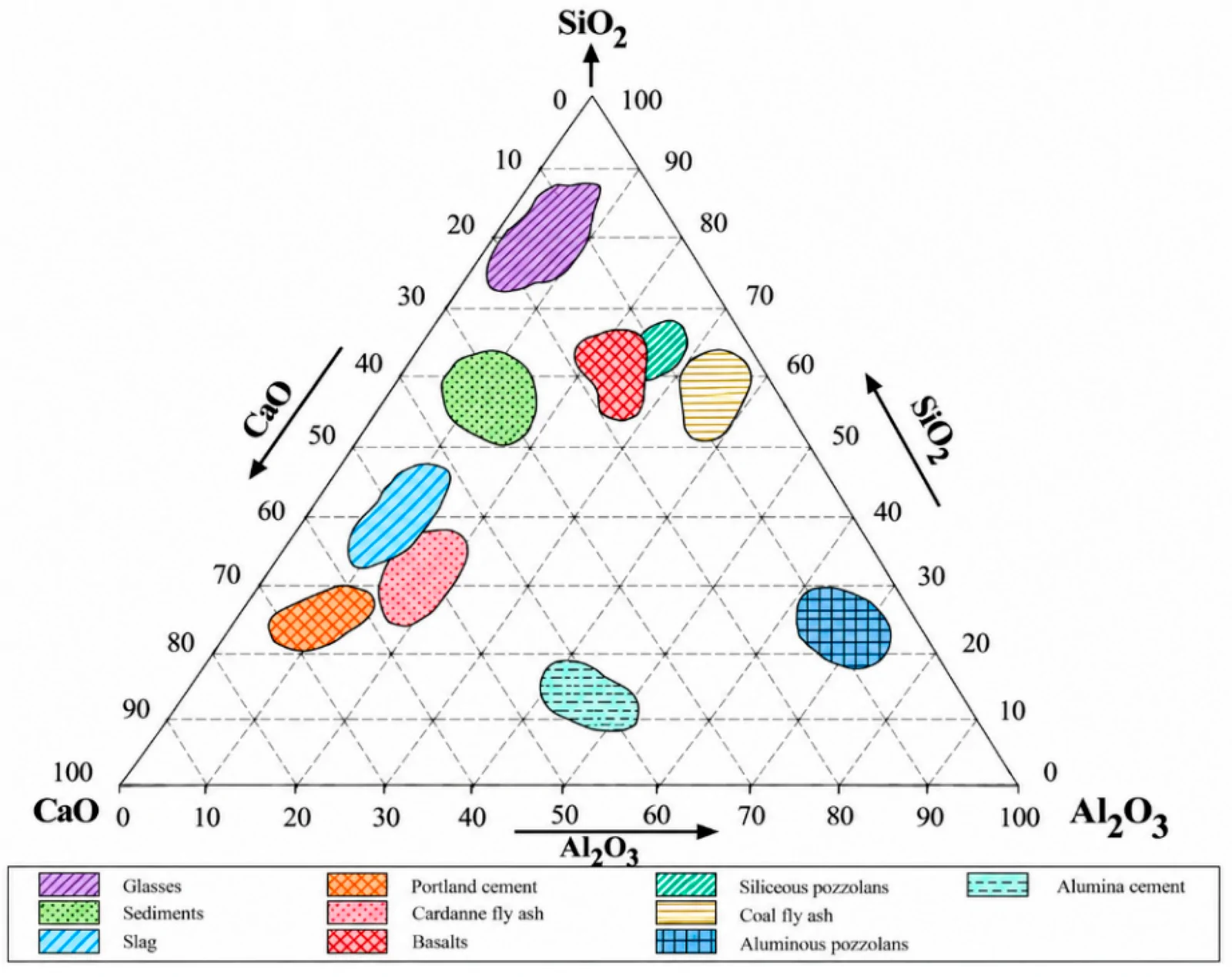
Sediment Classification in the Keil-Rankine Diagram based on their oxide composition (SiO_2_–Al_2_O_3_–CaO), adapted from [70].

The chemical compositions can be further interpreted using the ternary diagram of fine-grained materials shown in Figure 7. The diagram positions various materials based on their oxide compositions, specifically SiO_2_, Al_2_O_3_, and CaO. Sediments are compared to other common cementitious and pozzolanic materials such as Portland cement, slags, fly ash, alumina cement, and natural pozzolans. Sediments are positioned near the center of the diagram, with a slight excess of silica (SiO_2_) and a moderate balance of alumina (Al_2_O_3_) and lime (CaO), placing them between natural pozzolans, slags, and glasses. Compared to conventional binders, such as Portland cement (rich in CaO) and alumina cement (rich in Al_2_O_3_), sediments have intermediate properties. Their position reveals their hybrid chemical nature, suggesting potential reactivity when incorporated into cementitious materials and when combined with lime or industrial by-products [70].

Recent studies have demonstrated that the relative proportions of SiO_2_, Al_2_O_3_, and CaO largely determine the most appropriate valorization pathway for dredged sediments. Amar et al. [42] showed that sediments rich in reactive silica and alumina exhibit a greater potential for pozzolanic or geopolymeric applications, whereas sediments containing higher calcium contents may develop hydraulic reactivity when combined with activators. Similarly, Ma et al. [12] demonstrated that the chemical composition of dredged sediments governs not only their reactivity but also their compatibility with different construction materials, including cementitious binders, fired ceramics, and alkali-activated materials.

In addition, significant differences in oxide composition may occur even among sediments originating from similar geographical regions, as reported by Solanki et al. [13]. This can result from the heterogeneous chemical characteristics controlled by local geological formations, industrial activities, and sediment transport processes. Martellotta et al. [21] noticed considerable variability in both the chemical and mineralogical composition of sediments collected from different reservoirs, highlighting the need for comprehensive characterization before any reuse strategy can be proposed.

These findings emphasize that the suitability of dredged sediments for construction applications, including brick manufacturing, should be assessed on a site-specific basis, as the balance between silica, alumina, and calcium strongly influences firing behavior, mineral transformations, and the properties of the final ceramic products.

The mineral components primarily influence the physical and mechanical properties of sediments. Typically, sediment particle diameters range from 2 to 63 μm, corresponding to the clay and silt fractions, as per the French Road Earthworks Manual (GTR guide) [71]. According to Dubois [72], the finer fractions of sediments are responsible for many critical properties and exhibit complex behaviors. These fine particles demonstrate cohesive properties and possess a large, positively charged surface area, which enhances their capacity to adsorb contaminants.

Table 3 presents the grain sizes of various marine sediments, providing an overview of the general sediment granulometry. Significant variability is observed, largely influenced by the origin of the dredged sediments and generally indicates a considerable quantity of fine materials.

**Figure 8:**
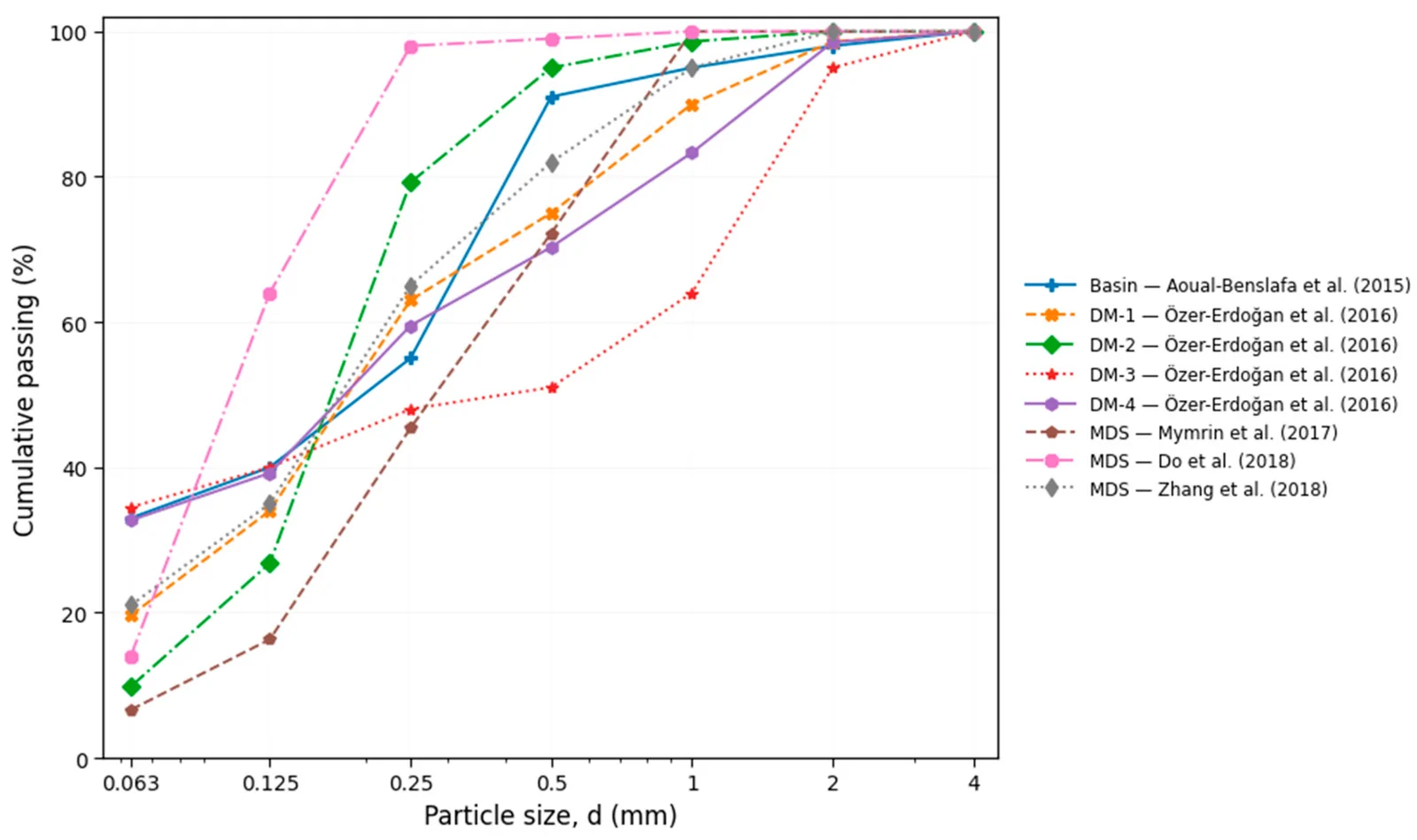
Particle size distribution curves of dredged sediments reported in the literature. Data were compiled by Chu et al. [82] from Aoual-Benslafa et al. (2015) [83], Do et al. (2018) [84], Mymrin et al. (2017) [30], Ozer-Erdogan et al. (2016) [60] and Zhang et al. (2016) [85].

Figure 8 illustrates the particle size distribution curves of various marine sediments dredged from four different continents: Africa (Algeria), South America (Brazil), Asia (South Korea), and Europe (Ireland and Turkey). These data were compiled from the literature by Chu et al. [82], including works by Aoual-Benslafa et al. (2015) [83], Do et al. (2018) [84], Mymrin et al. (2017) [30], Ozer-Erdogan et al. (2016) [60], and Zhang et al. (2016) [85]. One can notice the variability in sediment composition, with most studies indicating a predominance of fine particles, as discussed previously.

### 2.3. Challenges Related to Dredged Sediment Management

The management of dredged sediments presents many challenges, including environmental, technical, regulatory, logistical, and socio-economic dimensions. A major issue is sediment contamination, as dredged materials often accumulate significant quantities of heavy metals (such as lead, cadmium, and mercury) [19], organic pollutants (including PAHs, PCBs, and TBT) [86], and nutrients like phosphorus and nitrogen originating from industrial discharges, agricultural runoff, and urban wastewater [87]. These contaminants, predominantly found in the fine sediment fractions, pose risks to both ecological and human health [88]. As a result, dredged materials often require physico-chemical characterization and stabilization or decontamination before being considered suitable for beneficial reuse, particularly in construction.

It should be noted that the concentration and nature of contaminants in dredged sediments cannot be generalized according to geographical region alone. Even sediments collected from different ports within the same country, or within the same harbor, may exhibit markedly different physicochemical characteristics due to variations in watershed geology, industrial activities, navigation intensity, historical pollution, sediment granulometry, sampling location, and dredging conditions.

Moreover, the presence of organic matter further complicates sediment valorization. Organic components, particularly humic and fulvic acids, may inhibit cementitious reactions by neutralizing alkaline conditions required for binder activation [89]. This can make hydraulic binders less effective, delay strength development, and negatively influence the consistency and durability of sediment-based construction material [90]. The biodegradability of organic matter may also pose long-term durability concerns, including shrinkage, porosity, and water retention issues [91].

Another critical issue is volume management. Globally, the quantities of dredged sediments are significant, exceeding 350 million cubic meters annually in the USA and China, and tens of millions of cubic meters in Europe (CEDA-2022 [92]). Sediments are mainly removed to ensure navigation of harbors, estuaries and inland waterways. However, the buildup of such volumes places significant pressure on storage capacities, sediment management infrastructure, and final disposal approaches. The lack of centralized facilities and high costs of transport and processing often delay the development of regional or national reuse plans.

Regulatory complexity is another significant barrier. Various countries enforce their own national laws using the international framework established by the OSPAR Convention, Barcelona Convention, and London Protocol that deal with issues related to dredging and dumping. In France, the Environmental Code and the classification of ICPE require that sediments be assessed and classified based on contaminant thresholds. Depending on the classification, reuse options may be severely constrained, whether inert or hazardous. Ambiguities in regulation, evolving standards, and technical setbacks could disrupt project planning and discourage investment in reusing sediment.

The challenges are not only technical but also socio-economic and perceptual. Dredged sediments are often considered waste, eliciting opposition from communities, local governments, and even end-users. Such social considerations affect public acceptance and market integration, regardless of technical compliance and environmental safety. In addition, traceability requirements, legal liability concerns, and uncertain market value limit private sector engagement. Stakeholders often face procedural obstacles during public consultations or environmental permitting processes, especially when attempting to implement reuse strategies such as land reclamation or construction material production.

## 3. Sustainable Approaches for the Valorization of Dredged Sediments in Brick Production

Over the past years, numerous studies have explored the potential of utilizing dredged sediments as a sustainable and cost-effective alternative raw material in various civil engineering applications. These sediments are particularly used in the manufacturing of cement, concrete, bricks, paving blocks, mortar and road construction.

This section provides a literature review of the application of sediment in the production of bricks and blocks, which constitutes the primary focus of this review paper.

Brick is recognized as one of the most widely used construction materials, particularly in constructing buildings’ outer and inner walls. Annually, 1500 billion bricks are produced around the world. Of these, 1300 billion bricks (or 87%) are produced in developing countries [93]. Due to the high demand for raw materials by the construction industry, especially for producing bricks and blocks, researchers are exploring alternative sources. In this context, the reuse of dredged sediments, which are abundant and inexpensive, represents a sustainable solution to address both environmental concerns and resource scarcity, while also contributing to the valorization of these materials. Numerous research studies have investigated the possibility of using river and marine sediments in producing different types of bricks and blocks as the primary raw material or as a partial substitute for sand or other materials. Some concentrate on the production of fired bricks [56,63,65,93,94,95], while others focus on unfired alternatives such as compressed earth bricks [96,97,98,99] and Adobes [80]. Additionally, other investigations consider their application in concrete or paving blocks [100,101,102,103].

The shaping technique adopted for brick production also influences the energy requirements and overall sustainability of the manufacturing process. Extrusion and compression molding are the two predominant forming techniques for sediment-based bricks, each involving distinct processing conditions. Extrusion is predominantly employed for the manufacture of fired bricks, where plastic mixtures with relatively high moisture contents are required to ensure adequate workability and continuous shaping, resulting in greater energy consumption during the forming and drying stages. In contrast, compression molding is commonly adopted for compressed earth blocks (CEBs), adobe bricks, and paving blocks, where relatively drier mixtures are compacted under high pressure, thereby reducing water demand and energy consumption during shaping. It can be noted that the overall environmental performance of sediment-based bricks depends on the entire production chain, including sediment pretreatment, dewatering, drying, and thermal treatment, rather than on the shaping technique alone [12,14,22].

Researchers have examined the integration of dredged sediments into bricks at both laboratory and industrial levels. Table 4 summarizes the main studies on this topic, which show the origin of sediment, the percentage of incorporation, the brick types, the shaping technique and the experimental scale. The detailed physicochemical characteristics of the sediments, with the resulting physical, mechanical, thermal, durability, and environmental performances, are subsequently presented and critically discussed in the upcoming sections.

Industrial-scale studies, such as those using 50% Bremen harbor sediment (Germany) and 45% stabilized sediment from northern France, have successfully produced bricks meeting all technical and environmental standards [62]. Samara et al. [58] conducted one of the first large-scale industrial trials by replacing 15% of quartz sand with river sediments treated via the Novosol^®^ process, a stabilization method combining phosphatation and calcination to immobilize heavy metals and remove organic matter. A French brick factory manufactured around 15,000 bricks with standard equipment in the country. The resulting bricks exhibited 63% improvement in compressive strength, reduced porosity and water absorption, and only a slight increase in firing shrinkage. Similarly, El Fgaier et al. [107] evaluated bricks containing 15% treated sediment in real-world conditions by building and monitoring a 20 m^2^ test building in northern France. The compressive strength of sediment-amended bricks was found to be superior (36 MPa against 22 MPa for the conventional bricks). They showed reduced water absorption and better thermal resistance (0.36 m^2^·K/W against 0.33). The industrial-scale experiment demonstrated that we could use bricks made from sediments without imposing any environmental constraints. The main challenge is to obtain material with low moisture content, so that it does not swell when heated up, and thus prevents cracking of the bricks.

At the laboratory scale, the use of marine sediments in producing fired bricks has been investigated by many researchers. For instance, research conducted by Lafhaj et al. [59] investigated the possibility of integrating polluted river sediments from the North region of France at different percentages. These sediments, characterized by high concentrations of heavy metals and organics, underwent treatment using the Novosol process. Different blend designs were analyzed, featuring sediment ratios up to 45%. The findings confirmed the feasibility of utilizing treated river sediments to replace sand and clay in the manufacturing of bricks. Significantly, the substitute ratio of 35% was found to be the most effective in terms of physical, mechanical and chemical properties of the materials. A study by Slimanou et al. [63] examined the potential use of harbor dredged sediments as raw materials in the manufacture of fired clay bricks. The findings revealed that incorporating 15–20 wt% harbor-dredged sediments in brick enhanced its physical, mechanical, and thermal properties compared to control bricks.

**Figure 9:**
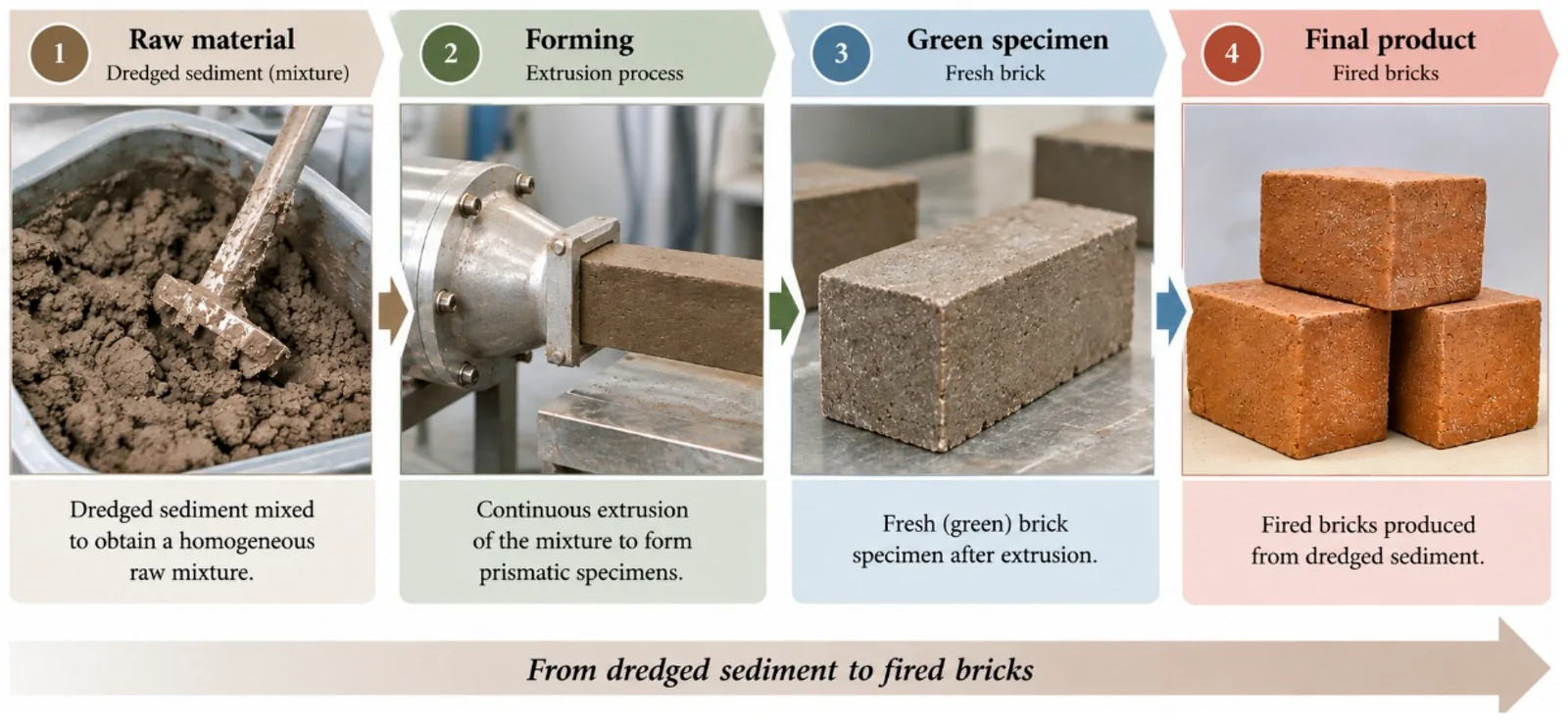
Schematic representation of the production process of fired bricks incorporating dredged sediments.

Xu et al. [66] investigated the possibility of utilizing urban river sediments from the Qinhuai River, China, to manufacture high-insulation bricks. Findings revealed that bricks containing 50% sediment and fired at 1050 °C exhibited favorable characteristics, including lower thermal conductivity and adequate compressive strength. Mezencevova et al. [56] utilized sediments dredged and extracted from Savannah Harbor, as illustrated in Figure 9. The study tested two brick compositions: one with 100% dredged sediment and the other with a combination of 50% sediment and 50% clay soil. When fired at a temperature of 1000 °C, these bricks achieved compressive strength meeting the ASTM standard for the lowest grade building bricks (NW). The addition of natural clay into the sediment significantly enhances its compressive strength. Bricks fired at 1000 °C achieved an average compressive strength of 29.4 MPa, exceeding the requirements for severe weathering (SW) building bricks.

**Figure 10:**
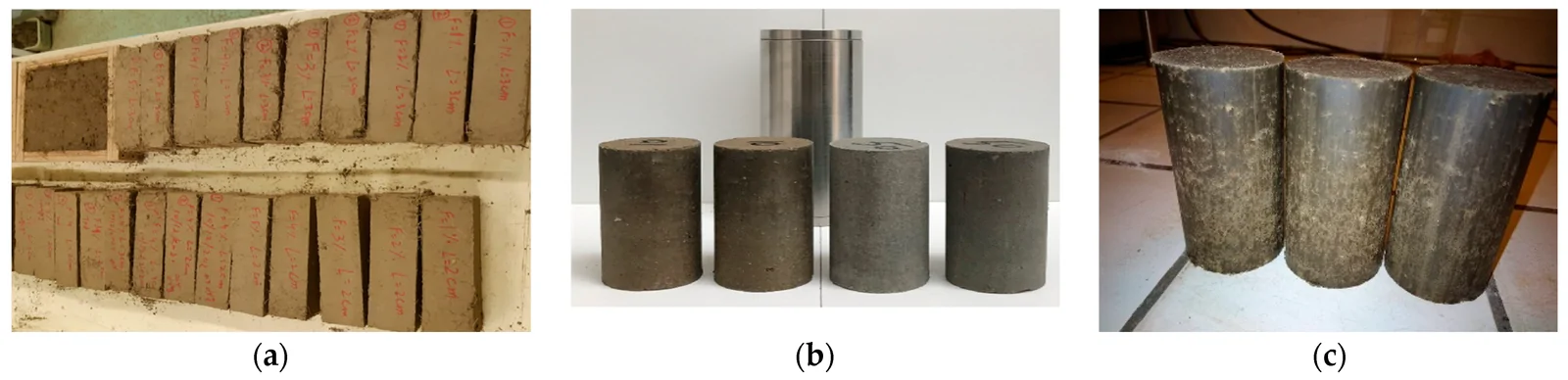
Unfired fabricated sediment-based earth bricks. (**a**) Adobe [106]; (**b**) CEBs [98]; (**c**) CEBs [97].

The application of marine sediments in the production of unfired bricks has also been explored (see Figure 10). For instance, Hussain et al. [106] explored the feasibility of incorporating sediments from the Dunkirk Port (France) and Usumacinta River (Mexico) in adobe brick production. The sediments were mixed with natural fibers, hemp shiv and palm oil flower fibers to improve the mechanical properties of the bricks. Findings showed that the incorporation of dredged sediments and natural fibers in the formulation of adobe bricks is a suitable solution for producing bricks with acceptable mechanical performance.

In recent years, dredged sediments have attracted increasing interest in the manufacturing of Compressed Earth Blocks (CEB) due to their abundance, mineralogical compatibility with earthen materials, and potential to reduce dependency on raw soils. A study conducted by Nassar et al. [99] assessed dredged sediments from Arcachon Bay (France) for their suitability for CEBs. Comprehensive characterization, including artisanal tests performed in the field and standardized geotechnical and mineralogical tests, revealed high clay and organic matter levels. While these properties enhance the plasticity and workability of sediments, they also present challenges such as increased drying shrinkage and reduced mechanical strength, particularly in sediments with high fine particles. As most samples did not comply with the recommended criteria for CEBs, stabilization strategies are proposed. These strategies included the physical addition of sand to modify particle size distribution, thus reducing plasticity and stabilization involving chemical geopolymerization to enhance structural integrity. In addition, a machine learning model was trained on literature data on sediment composition to predict compressive strength, which provides a new tool for efficient mix design and performance prediction. The research concluded that, with proper treatment and optimization of the formulation, these sediments can be considered a sustainable and technically feasible raw material for CEBs.

According to Larbi et al. [110], using dredged sediment as a constituent for CEB production, along with 10% furnace slag and 5% glass, meets the recommended specifications for stress and water resistance of various standards.

Brahim et al. [98] investigated the use of dredged sediments from Northern France as main raw materials to produce CEB, stabilized with alkali-activated fly ash. They substituted 10–50% of the sediment with fly ash and activated it with sodium hydroxide. An optimal substitution in the range of 10–20% enhanced mechanical and physical properties significantly. The study concluded that such stabilized CEBs meet current construction standards and offer a sustainable alternative to traditional materials. Serbah et al. [96] studied the use of dredged sediments of the Bakhadda dam (Algeria) in CEBs. Sand was added, in proportions of 10 to 30%, to meet standard granulometry and plasticity requirements. Specimens compacted at an energy level higher than that of the Modified Proctor test exhibited improved mechanical performance. The study also emphasized the strong influence of water content on CEB strength.

The study conducted by Belayali et al. [97] assessed the feasibility of dredged sediments from northern France as a sand substitute for compressed earth blocks (CEBs) stabilized with fly ash (FA) and ground blast furnace slag (GBFS) geopolymers. The results demonstrated that incorporating 36% fly ash and 10% GBFS, activated with a sodium hydroxide (NaOH) alkaline solution, significantly enhanced the mechanical properties of the CEBs.

Although the basic guidelines for earthen construction are still limited and fragmented, the technical recommendations are emerging. Brahim et al. [98] suggest that suitable soil for CEBs should have at least 5% clay (as natural binder) and not exceed 15% to avoid shrinkage cracking. The plasticity index should ideally range between 15 and 30, and dry density should fall between 1600 and 2200 kg/m^3^ [111,112]. In addition, particle size should not exceed 10 mm [113,114].

**Figure 11:**
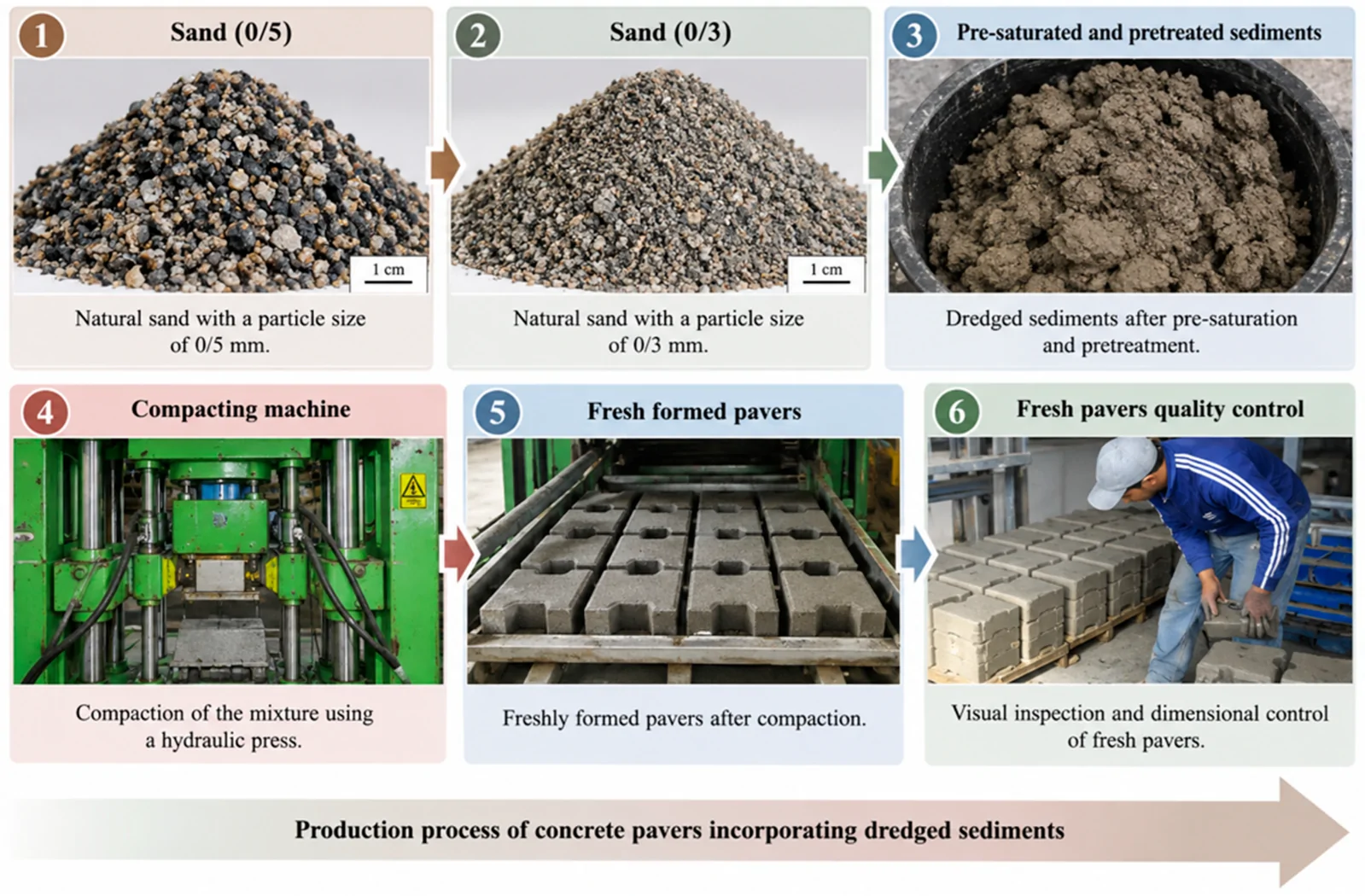
Main materials and production stages of concrete pavers incorporating dredged sediments.

Beyond traditional bricks, dredged sediments have also been utilized in the production of pavement blocks. According to Said et al. [100], non-polluted sediments of Rades harbor could be reused in the production of paving blocks (see Figure 11). In this study, sediments were utilized to replace sand 0/3 (quartz sand) with a substitution ratio of 19%. Subsequently, a full-scale industrial experiment was conducted at a paving block factory in northern Tunisia. The results indicated that the produced paving blocks meet specifications and environmental standards.

In a study conducted by Wang and colleagues [28], contaminated sediment was transformed into eco-friendly paving blocks through binary cement and carbon dioxide curing. Wang et al. [101] also developed efficient and value-added methods to convert contaminated sediments into fill materials, partition blocks and paving blocks. The findings reveal technologically feasible, environmentally responsible, and potentially profitable waste management solutions for dredged sediment.

## 4. Influence of Dredged Sediments on Brick and Earth Blocks Production

Bricks have been a fundamental building material for thousands of years, since at least 8000 B.C. [115], and their demand continues to grow. Global brick production is estimated at approximately 1.83 trillion units per year and projected to rise to 2.76 trillion units by 2027, driven by a 3% annual growth in the building sector [92]. However, the traditional brick industry is highly resource- and energy-intensive, consuming large quantities of raw materials and contributing significantly to environmental pollution [116].

As part of efforts to improve sustainability in the construction sector and advance the development of low-carbon building materials, particularly bricks and blocks, which are widely used in the construction industry, one promising and responsible approach is the use of waste as a raw material. In this regard, dredged sediments, due to their abundant availability and favorable mineralogical composition, offer a promising alternative resource for brick production. Several studies have been conducted in this area, and the results indicate that dredged sediments can often be incorporated into bricks at different proportions [58,59,62,63,96,110]. Such measures encourage circular economy strategies while significantly reducing environmental impact.

Numerous studies explore the technical feasibility of using river, lake, marine and reservoir sediments in the making of bricks and blocks. This includes fired clay bricks, adobe bricks, compressed earth blocks (CEBs), geopolymer- and cement-stabilized earth blocks, and paving blocks. These investigations generally examine the quality of bricks and blocks resulting from dredged sediment usage. Within this section, a detailed literature review will examine the influence of incorporating dredged sediments on the physical properties, mechanical strength, thermal properties and the environmental performance of bricks. Each of these aspects will be discussed separately in dedicated subsections.

It should be noted that the studies reviewed in this section investigate different categories of sediment-based masonry materials. These materials differ fundamentally in terms of raw materials, manufacturing processes, bonding mechanisms, curing or firing conditions, and applicable technical standards. Therefore, the physical, mechanical, thermal, durability, and environmental properties reported in the literature should not be interpreted as direct quantitative comparisons between these material categories. The results are discussed to evaluate the influence of dredged sediment incorporation within each material system and to identify the principal factors governing their performance.

### 4.1. On Physical Properties

The incorporation of dredged sediments into brick formulations significantly affects key physical parameters that play critical roles in shaping the final quality, durability, and processability of the materials. Table 5 presents the results of previous studies conducted on the physical properties of formulated bricks.

The bulk density is a primary indicator of brick compactness and microstructure and is influenced by the raw material and the firing procedure. Slimanou et al. [63] demonstrated that replacing natural clay with dredged sediments decreased the bulk density of fired bricks at 850 °C and 950 °C temperatures. Reference bricks with 100% clay had densities of 1841 kg/m^3^ and 1854 kg/m^3^, respectively. Bricks with 100% sediment exhibited slightly lower bulk density values of 1778 kg/m^3^ and 1771 kg/m^3^. This decrease was primarily attributed to the finer particle size and lower inherent density of the sediment material.

Xu et al. [66] have reported that the use of urban river sediments causes a reduction in bulk density to the level of 1210 and 1450 kg/m^3,^ which is considerably lower than the levels of fired bricks that reach up to 1800 and 2000 kg/m^3^. It was noted that as the sediment content increased, the densities decreased at all firing temperatures. Notably, bricks fired at 1050 °C exhibited more densification due to optimal thermal energy promoting sintering. However, firing at 1100 °C led to mechanical expansion because of the excessive internal gas pressure in closed pores. The substantial reduction in density occurs due to the rapid expansion of these structural defects. This is a phenomenon associated with over-firing [117].

Similarly, Ducman et al. [109] observed a consistent drop in density with increasing sediment content. Bricks prepared with pure clay (0% sediment) demonstrated a density of 1760 kg/m^3^, while bricks prepared with 50% sediment showed a reduced density of 1520 kg/m^3^. The drop was directly related to the greater organic and carbonate contents of the sediments, which decompose on firing, causing further porosity and lowering the overall mass-to-volume ratio.

In contrast, Chiang et al. [104] obtained different results when they evaluated the feasibility of reservoir sediment as a principal raw material. Bricks made up of 80–100% sediment were significantly densified at high temperatures of 1100 °C, as evidenced by the bulk density of nearly 2500 kg/m^3^ for the 100% sediment (0% clay) brick. This suggests that, under appropriate thermal conditions, certain sediment types can completely substitute traditional clays while maintaining structural compactness.

In parallel, the effect of sediment addition on loss on ignition (LOI), a measure of volatile content and thermal stability, was also examined. Xu et al. [66] observed that increasing sediment content increased the LOI by 9.13% to 11.73% with firing temperatures of 1000 °C to 1100 °C. These values align with typical porous bricks [118]. Thus, organic and carbonate-rich sediments do not weaken the material’s quality.

Mezencevova et al. [56] confirmed these findings through a study of bricks made from either 100% dredged sediment or 50:50 sediment-clay blends. All the bricks tested remained below the 15% LOI threshold specified by AASHTO (1982), indicating that mass loss due to the combustion of organics and volatiles was within acceptable limits. The results indicate that the presence of dredged sediment did not adversely affect the stability of the bricks during thermal processing and is compatible with industrial firing conditions.

In addition, plasticity, which plays an important role in shaping and handling raw brick mixtures, was also affected by sediment incorporation. According to Lafhaj et al. [59], increasing the proportion of treated dredged sediment reduced the plasticity index (PI) of the mix, which in turn lowered its cohesion and bonding strength. The resulting mixtures were classified as having low plasticity. While it may be beneficial to reduce shrinkage and cracking during drying, additional additives or stabilizers may be needed to restore optimal workability and mechanical performance during forming.

Another important property of bricks to be evaluated is water absorption. In fact, water absorption is an essential property that can influence the durability and service life of construction materials. It is directly correlated to open porosity and the material’s ability to resist environmental degradation. Higher water absorption enables moisture and aggressive agents such as chlorides, sulfates and frost to penetrate concrete, which destroys the concrete through efflorescence, cracking and freeze–thaw damage. On the other hand, low water absorption is indicative of a dense and cohesive microstructure, which enhances the durability of the concrete under changing weather conditions. The integration of dredged sediments into brick and compressed earth block (CEB) formulations affects water absorption behavior, depending on the sediment type, firing temperature, substitution ratio, and the presence of stabilizing agents.

Lafhaj et al. [59] measured the water absorption as per the French standard [119]. The authors noted that all tested mixtures remained within the regulatory threshold of 40%. However, the water absorption of the permeable pavement increases with an increase in sediment content. There are two main factors for this increase. First, the addition of treated sediment tends to lead to a reduction in the cohesiveness of the clay matrix and an increase in the internal pore volume, allowing better water retention ability. Second, the calcite (CaCO_3_) content of the sediment affects its porosity development during the firing process. At firing temperatures greater than 800 °C, calcite decomposes into calcium oxide (CaO), which induces microporosity in the structure. The reaction between CaO and water vapor produces portlandite (Ca (OH)_2_) and exerts crystallization pressure in the pores. This pressure can potentially induce microcracking and further increase porosity and water absorption.

Xu et al. [66] reported similar observations for bricks prepared with an urban river sediment content of 50–70%. Water absorption increased with sediment content while it decreased when firing temperature was raised from 1000 °C to 1050 °C. At this temperature, densification transformed open pores into closed pores due to particle rearrangement, thereby altering the overall pore structure of the brick. However, at the highest firing temperature of 1100 °C, defects like cracks, blisters and bloating were observed, indicating structural degradation. The water absorption was enhanced due to disruption in the porosity network caused by defects. Conversely, fired bricks at the temperature of 1050 °C in the same formulations exhibited the lowest water absorption values, suggesting that this temperature offers an optimal balance between porosity control and structural integrity.

Furthermore, Mezencevova et al. [56] investigated the impact of firing temperature on the water absorption of fired bricks amended with sediments. Bricks were made with 100% and 50% dredged sediment. It was observed that with an increase in firing temperature, the water absorption capacity decreased, whereas, on the other hand, with an increase in initial moisture content, the water absorption capacity increased. Despite these variations, all bricks complied with ASTM C62 specifications for water absorption of bricks subjected to severe and moderate weather conditions, confirming their structural suitability. Similarly, Chiang et al. [104] tested bricks containing 80–100% reservoir sediment; the authors observed that water absorption decreased by 80% as the sintering temperature increased from 1000 °C to 1100 °C. At temperatures above 1050 °C, all bricks met Taiwan’s water absorption standard (≤15%); this is attributed to the reduction in open porosity through enhanced vitrification and densification at high temperatures.

Ducman et al. [109] reported that increasing the sediment percentage from 0% to 50% caused the water absorption to rise from 17.2% to 25.1% because of organic matter and carbonate decomposition during firing. These combustion reactions generate gas release, pore formation, and matrix weakening, particularly at high substitution levels.

In contrast, a different trend was observed in some previous studies conducted on fired bricks. Slimanou et al. [63] confirmed that low sediment substitution percentage improved performance. At 850 °C and 950 °C, water absorption decreased with the integration of up to 20 wt% and 15 wt% harbor dredged sediment, with absorption values of 14.37% and 14.48%, respectively. However, above these proportions (50 and 100 wt%), water absorption increased, reaching 16.32% at 850 °C and 21.45% at 950 °C in bricks containing 100% sediment. These results correlate with open porosity measurements: control bricks had porosities of 30.9% (850 °C) and 28.5% (950 °C), while 20% sediment replacement reduced porosity to 27.3%. At higher sediment contents, porosity and water absorption increased, specifically at elevated temperatures.

**Figure 12:**
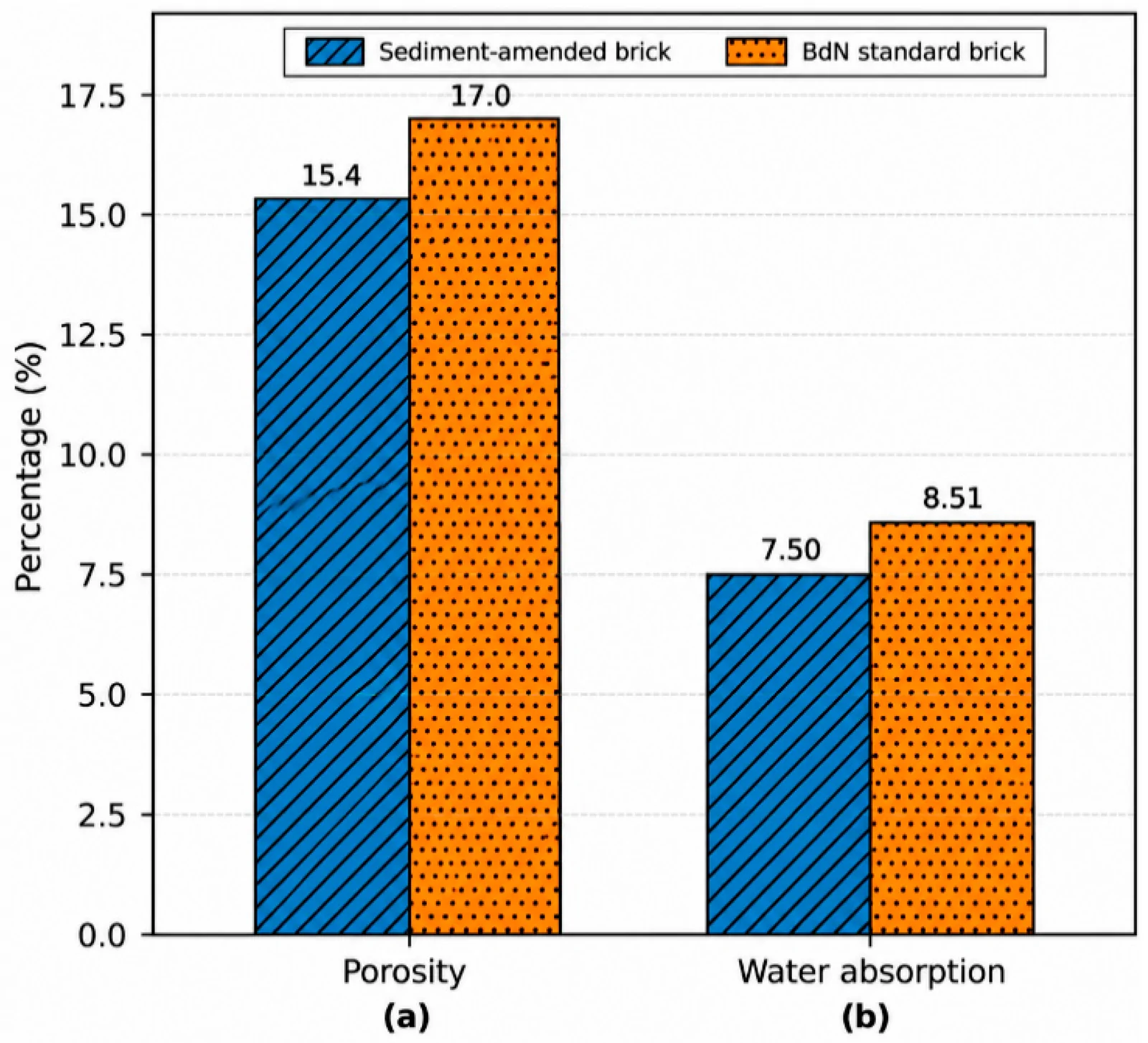
Porosity and water absorption of sediment-amended and BdN standard bricks, reconstructed from Samara et al. [58].

In addition, Samara et al. [58] demonstrated that replacing 15% of quartz sand with treated river sediment reduced both porosity and water absorption relative to conventional bricks (BdN), as seen in Figure 12.

The improvement was associated with the finer granulometry of the sediment, which improves solid-state sintering below 900 °C and vitrification at higher temperatures. During sintering, the particles are more closely packed. This results in smoother surfaces and more closed pores with ellipsoid shapes. Therefore, it reduces water penetration. This densification behavior was assisted by fine sediment particles, which sinter faster and more uniformly than coarser aggregates.

**Figure 13:**
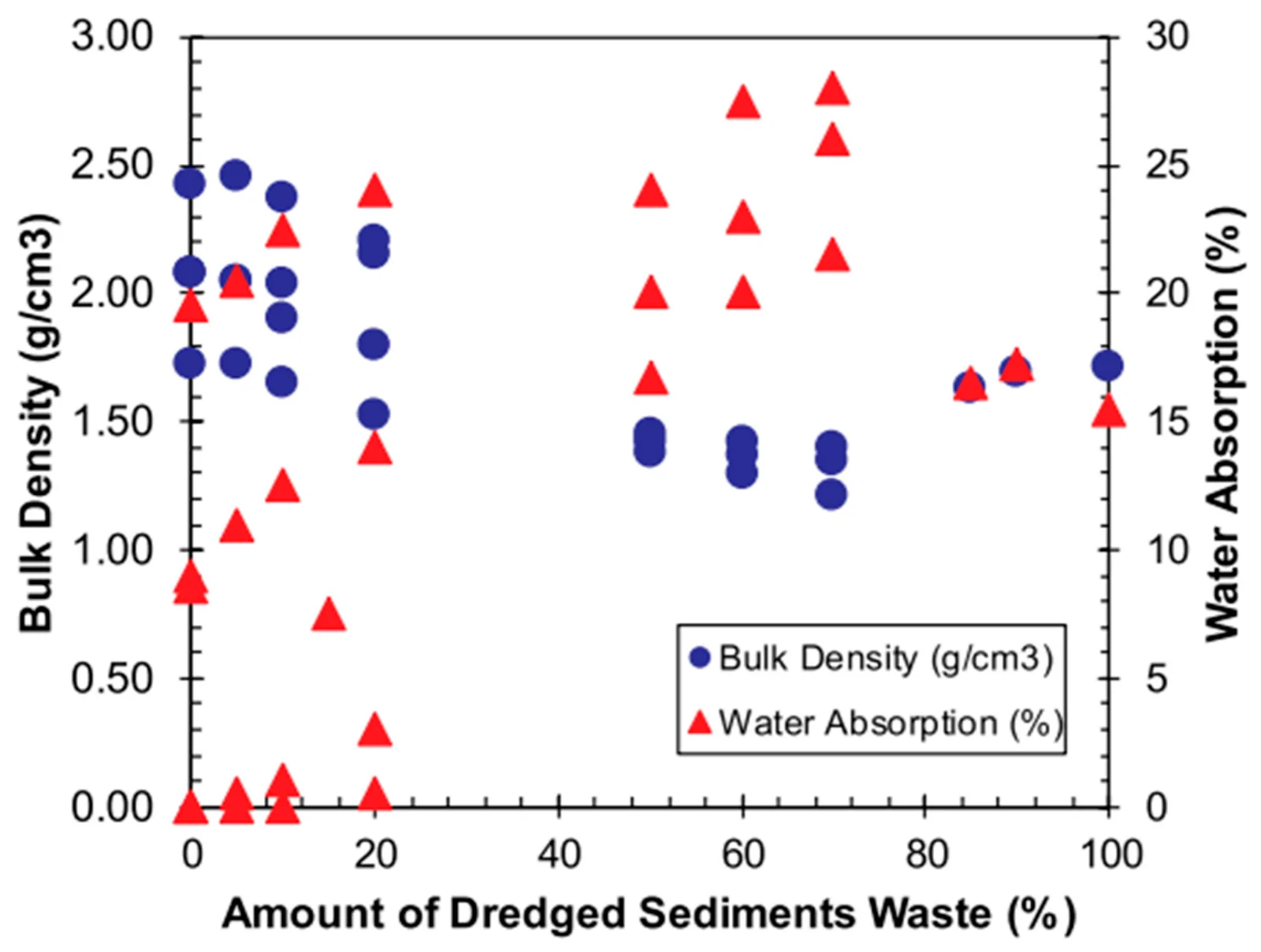
Effect of dredged sediment percentage on bulk density and water absorption of bricks [58,65,66,104].

Figure 13 summarizes the findings of bricks’ bulk density and water absorption of four studies mentioned previously. It can be seen that up to 30% of dredged sediment is beneficial for the production of bricks.

Beyond fired bricks, several studies have evaluated the effect of dredged sediments on the water absorption of unfired compressed earth blocks. Bhairappanavar et al. [57] reported that the water absorption of cement-stabilized sediment bricks was limited by cement content and compaction pressure. Bricks containing 8% cement without compaction absorbed 17.92% water, while bricks composed of 12% cement and compacted at 4 MPa absorbed only 13.73%. This indicates that cement acts as a binder and a filler for pores. Similarly, compaction is a structural densifier. Both reduce pore size and improve water resistance. All tested compositions comply with ASTM C67 specification for water absorption of ≤20%.

Brahim et al. [98] investigated CEBs manufactured with dredged sediment from Northern France and stabilized through activated fly-ash binders. The authors observed that as the fly ash content increased, the coefficient of water absorption by capillarity decreased. All stabilized samples met the French XP P13-901 [113] Standard for low-capillary materials, qualifying them for exterior wall applications.

In addition, Larbi et al. [110] investigated compressed earth blocks containing two different types of sediments from Algerian ports with 10% of blast furnace slag and different proportions of dissolved glass in a NaOH solution. The findings showed that blocks containing 4% glass powder activated with a 4 M NaOH solution demonstrated the lowest water absorption values, with a capillarity coefficient (Cb) being 16.2 g/cm^2^·min^0.5^ for 30 g of glass and 28.7 g/cm^2^·min^0.5^ for 40 g of glass. These values indicate improved compactness and reduced porosity compared to other formulations, where both lower (2%) and higher (6–8%) glass contents resulted in higher absorption. This suggests that excessive or insufficient glass powder disrupts optimal geopolymer gel formation. In addition, total water absorption and porosity measurements confirmed that the 4% glass/NaOH combination indicated better matrix densification.

It is important to mention that the differences reported in the literature regarding the physical properties of sediment-based bricks are primarily governed by the combined effects of sediment characteristics and manufacturing conditions rather than by sediment incorporation alone. Sediments with finer particle-size distributions generally promote densification and lower water absorption through improved particle packing, whereas high organic matter and carbonate contents increase gas evolution during firing, leading to higher porosity, lower bulk density, and higher water absorption. Likewise, the sediment incorporation ratio strongly influences the microstructure of the final product, with moderate replacement levels often improving physical performance, while excessive substitution may disrupt the ceramic matrix. Processing parameters, particularly the forming technique and firing temperature, further control pore development, vitrification, and densification, explaining the discrepancies observed among the reviewed studies.

Another crucial parameter to be discussed is the shrinkage of bricks. Shrinkage refers to the reduction in the dimensions of a brick during its manufacturing and results from two distinct mechanisms: drying shrinkage and sintering (firing) shrinkage. Drying shrinkage occurs during the initial drying stage before firing and is mainly caused by the evaporation of free and adsorbed water from the clay–sediment mixture. In contrast, sintering shrinkage develops during firing through particle rearrangement, solid-state diffusion, viscous flow, phase transformations and pore closure, which progressively densify the ceramic matrix. It directly influences the characteristics, mechanical strength, and structural compatibility. Shrinkage is a complex phenomenon involving physical and chemical processes like sintering, phase transformation and gas release. This phenomenon is sensitive to multiple factors such as raw material composition, particle size, moisture content and firing temperature.

The incorporation of dredged sediments influences both drying and sintering shrinkage, as these materials generally exhibit heterogeneous characteristics, including finer particle sizes, higher organic matter contents, higher water absorption capacity, and variable mineralogical compositions. Fine particles and clay minerals tend to increase the water demand during molding, thereby promoting drying shrinkage, whereas mineralogical transformations and the combustion of organic matter during firing significantly affect sintering shrinkage.

Slimanou et al. [63] indicated that all the bricks with harbor river sediments showed firing shrinkage lower than the 8% limit for conventional clay bricks [120] at 850 °C and 950 °C, respectively. Sintering at 850 °C and 950 °C causes significant transformations such as the combustion of organic matter, dehydroxylation of kaolinite, and decarbonation of calcite. The microstructure of the bricks is influenced by these reactions. At 850 °C, the amount of shrinkage was high when the sediment content was increased. There was a 0.67% shrinkage for 10 wt% substitution. The amount decreased to 0.19% for 50 wt%, though it increased again to 0.29% for 100% sediment. A different trend was seen at 950 °C, with the shrinkage decreasing from 1.29% for the control to 0.20% at 50% sediment, followed by a nearly constant value at 100%. These variations were linked to the quartz content, which forms the structural matrix of the bricks and mitigates shrinkage during firing. As the proportion of dredged sediments increases, the quartz content decreases; thus, the bricks’ structure becomes more susceptible to contraction, particularly evident in the 100 wt% sediment replacement at both firing temperatures.

Moreover, Samara et al. [58] examined the shrinkage in bricks where 15% of quartz sand was substituted by river sediment treated with Novosol^®^. The sediment-amended bricks showed higher shrinkage than reference bricks from a northern French factory (BdN). The increase was attributed to the finer particle size and lower quartz content of the sediment-based samples, which facilitated more intense sintering and densification. Additionally, the sediment–amended samples required approximately 2% more water to achieve the same plasticity level. The higher water demand generated greater drying shrinkage, whereas the finer particles and lower quartz content led to increased sintering shrinkage during firing, demonstrating that both mechanisms contributed to the overall dimensional changes.

Chiang et al. [104] produced brick containing 80–100% reservoir sediment content, with extra clay, and at different sintering temperatures. The shrinkage of the clay brick increased with clay content and firing temperature. The brick samples showed up to 32% shrinkage after firing at 1150 °C with 20% clay.

Furthermore, Mezencevova et al. [56] observed that the firing shrinkage of bricks made from 100% sediment ranged from 10.3% to 15.7%, exceeding the standard 8% threshold for good quality bricks [120]. According to the authors, the high moisture content of raw mixes, 32% to 47% as compared to ~19% typical for industrial extrusion, explains the obtained results. The sediment’s high organic content and fine particle size resulted in greater water absorption and retention during mixing. Consequently, more significant volumetric changes were observed during the drying and firing processes.

In contrast, the study of Ducman et al. [109] showed a different trend. Firing shrinkage of pure clay bricks was 1.3%, and that of dredged sediment bricks with 50% composition was 0%. This was attributed to the breakdown of carbonates and the release of gases that took place during sintering, which counteracted the typical contraction and contributed to dimensional stability. This behavior may reduce cracking and deformation during industrial-scale production.

Moreover, Xu et al. [66] showed that linear shrinkage in sediment-brick formulations exhibits a balance between densification and gas generation. Untreated clay bricks exhibited normal contraction behavior with linear shrinkage of 4.56%. Nevertheless, the addition of urban river sediments with high organic matter caused the microorganisms to expand rather than contract and thus release more gases. This phenomenon results in internal porosity and structural expansion, a behavior consistent with other studies on clay-waste mixtures [121,122]. The coexistence of sintering (which promotes densification and shrinkage) and gas release (which induces expansion) demonstrates the importance of thermal process optimization.

These studies demonstrate that the total shrinkage of sediment-based bricks results from the combined effects of drying shrinkage and sintering shrinkage. While drying shrinkage is primarily governed by moisture content, particle-size distribution, plasticity, and water demand, sintering shrinkage depends mainly on firing temperature, mineralogical transformations and gas evolution.

### 4.2. Influence of Dredged Sediments on the Mechanical Strength of Bricks

Brick compressive strength is one of the most critical parameters that determine the structural performance and suitability of bricks for construction applications. The mechanical strength of bricks can be affected by multiple parameters, such as cement content, water-to-cement ratio, hydration degree, compaction pressure, curing duration, and the nature of the coarse particle fraction [123]. Thus, understanding these variables is crucial to optimize brick formulation and achieve reasonable quality.

Numerous researchers have explored the effects of integrating dredged sediments into bricks and investigated the interactions between sediment content, firing temperature, and material composition. Most studies investigated the effects of sediments on fired bricks, with only a limited number having focused on the production of unfired earth blocks incorporating sediments. Table 6 summarizes the compressive strength results obtained for different studies. Because these materials differ in manufacturing processes, bonding mechanisms, and applicable standards, the reported values are discussed within the context of each material category rather than being directly compared.

**Figure 14:**
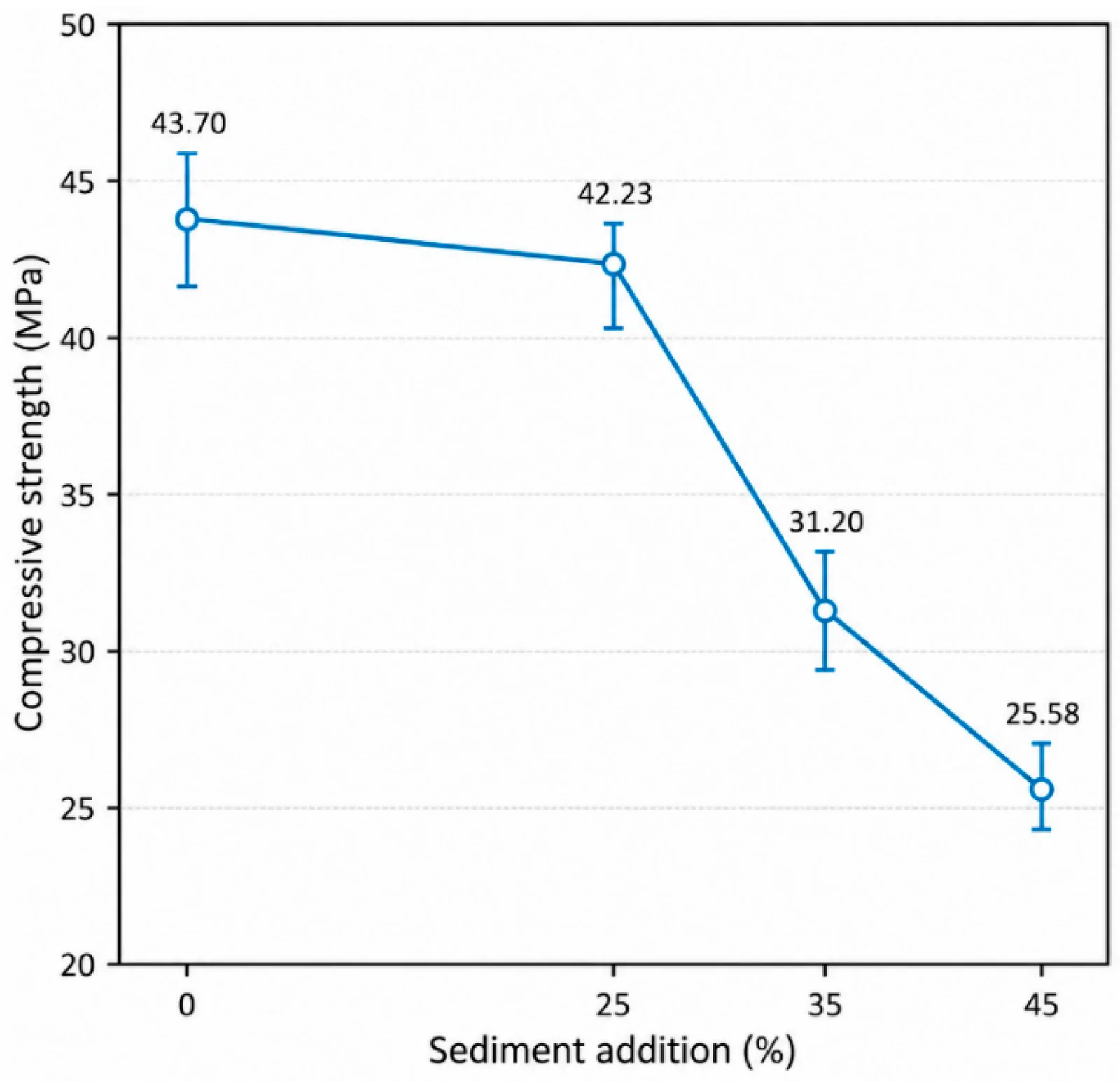
Effect of sediment addition on the compressive strength of fired bricks, reconstructed from data reported by Lafhaj et al. [59].

Lafhaj et al. [59] observed a general decrease in compressive strength with increasing sediment content in clay bricks (see Figure 14), due to increased internal porosity and reduced density caused by the sediments. The higher pore volume reduces the load-bearing capacity and thus the mechanical strength. The incorporation of 25% wt. of treated sediments leads to a compressive strength of 42.23 MPa, while a value of 25.58 MPa was observed for 45% sediment content. All the brick samples had compressive strengths comparable to standard bricks (18–20 MPa).

Similarly, Xu et al. [66] found that bricks with higher proportions of urban river sediments demonstrated lower strength values, due to weakening the material’s structural integrity. The authors also highlighted the positive role of firing temperature, showing that densification between 1000 °C and 1050 °C increases strength, with a 50% sediment brick fired at 1050 °C reaching 12.56 MPa.

In addition, Ducman et al. [109] reported a decrease in compressive strength of fired bricks incorporating sediment from Lake Ptuj. The reference sample, made from 100% brick-making clay, got a strength of 33 MPa. As the sediment content was increased, the compressive strength decreased gradually but was still in the acceptable range. A 50% substitution of sediments to clay produced a brick with a strength of 25 MPa. The reduced strength was associated with an increase in porosity due to the combustion of organic matter and carbonates existing within the sediment, which was reflected in the higher LOI and TOC values.

**Figure 15:**
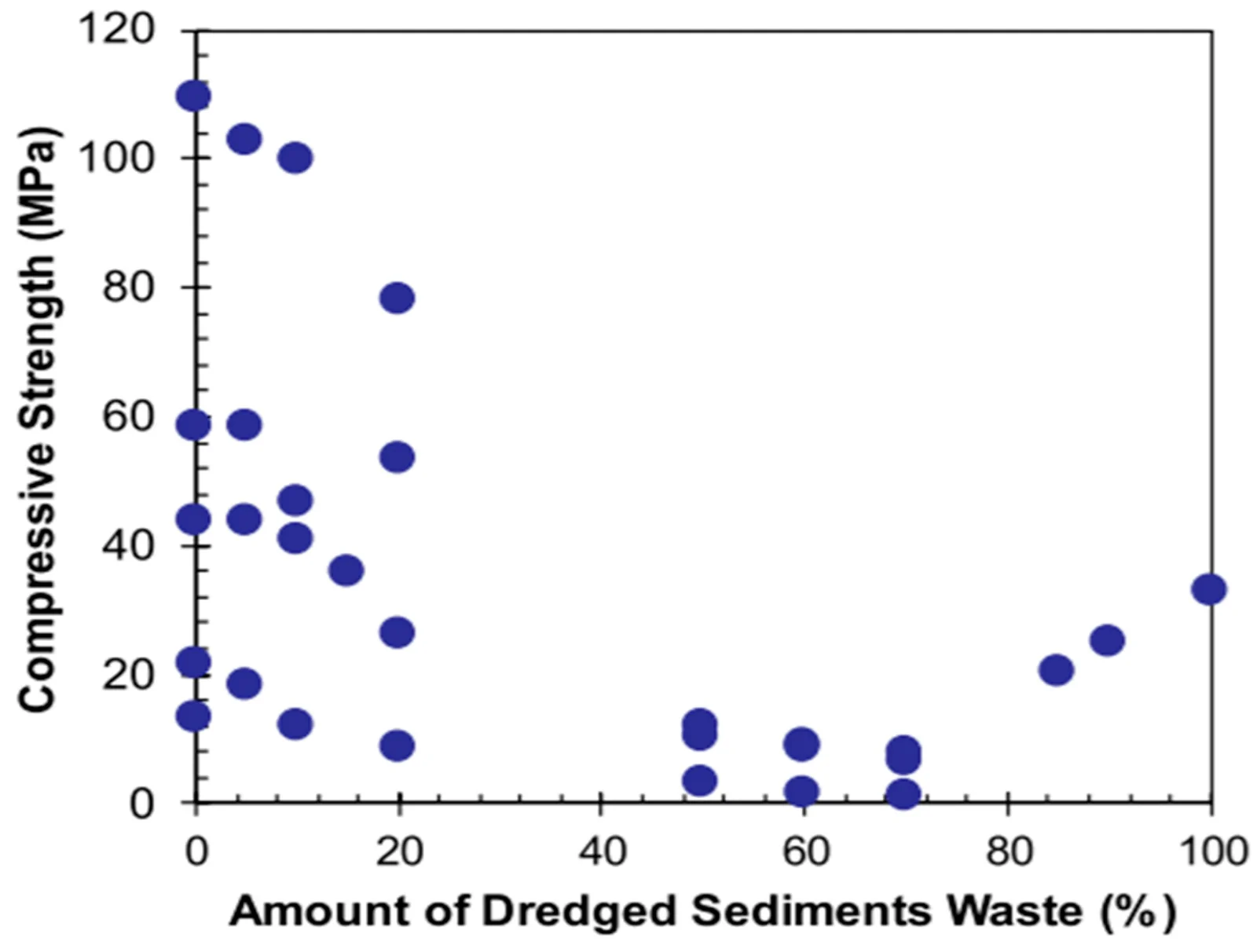
Influence of dredged sediments on compressive strengths of brick [58,65,66,104].

This trend can be noticed in Figure 15, which regroups the compressive strength results of some previous works. The mechanical properties are higher in bricks having lower sediment content.

**Figure 16:**
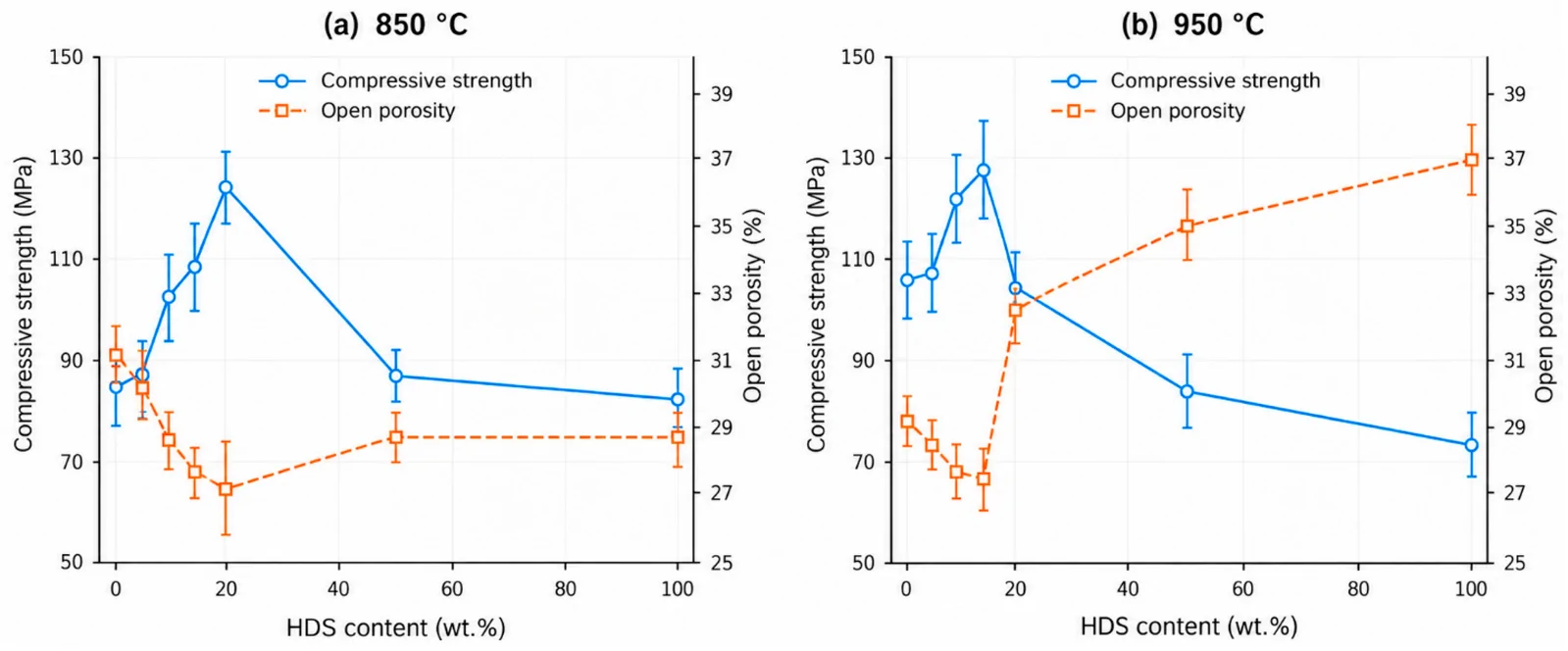
Effect of HDS content on the compressive strength and open porosity of fired clay bricks at (**a**) 850 °C and (**b**) 950 °C, reconstructed from data reported by Slimanou et al. [63].

In contrast, Slimanou et al. [63] demonstrated that harbor dredged sediments (HDS) can enhance the mechanical strength of fired bricks. The compressive strength of the bricks was significantly influenced by both the proportion of harbor dredged sediment (HDS) incorporated and the firing temperature. Control bricks made with 100% clay achieved compressive strengths of 84.3 MPa at 850 °C and 105.8 MPa at 950 °C, as can be seen in Figure 16. The addition of HDS up to optimal levels, 20 wt% at 850 °C and 15 wt% at 950 °C, led to an increase in compressive strength, reaching peak values of 108.2 MPa and 127.7 MPa, respectively. This improvement is likely due to the densification process occurring during sintering and the transformation of calcium aluminum silicate and diopside minerals. When the HDS content was increased beyond the optimal level, the strength was found to decrease.

The particle size and mineral composition of sediments significantly influence the mechanical strength of bricks. Samara et al. [58] confirmed a 63% improvement in strength when 15% quartz sand was replaced by treated river sediments, resulting in a finer and more homogeneous mix. The finer particle size enhances sintering during firing, resulting in a dense and more compact microstructure. This can be explained by density measurements, which show an increase from 1.99 g/cm^3^ for the standard bricks to 2.05 g/cm^3^ for the sediment-amended formulations. Quartz particles sized 10–30 µm contribute positively to strength, while larger grains may weaken the bricks.

Mezencevova et al. [56] compared the compressive strengths of fired bricks made solely from dredged sediments to bricks blended 50:50 with natural clay. The authors found that 1000 °C was the optimal firing temperature for achieving the highest strength in the bricks. In addition, bricks made entirely from dredged sediment and fired at this temperature met ASTM standards for the lowest grade (NW) building brick, with values ranging between 8.3 and 11.7 MPa. When clay was blended with the sediment, the resulting bricks reached an average compressive strength of 29.4 MPa, higher than 20.7 MPa, the minimum required for severe weathering (SW) grade bricks, demonstrating the enhanced structural performance achieved through this mixed formulation.

Hamer et al. [62] also confirmed the suitability of their bricks for non-load-bearing and general masonry applications. Bricks with 0% sediment, 40% clay, and 10% crushed recycled bricks have compressive strengths between 18 and 24 MPa. These values meet the requirements for standard building bricks under German construction standards.

Romero et al. [105] stated that bricks made with contaminated marine sediments from Astilleros, Cuchia and Raos (Spain) showed promising structural performance without treatment. The maximum compressive strengths achieved by the Astilleros and Raos sediments were found to be 34 MPa upon sintering at temperatures of 1125 °C and 1150 °C, respectively, while that of Cuchia sediments was found to be 11 MPa upon sintering at 1125 °C. The differences in strength among the samples were closely linked to mineralogical composition and sintering behavior. Astilleros and Raos exhibited greater densification and stronger vitrification, leading to higher mechanical resistance, while Cuchia’s lower strength was attributed to residual porosity and less efficient sintering.

Zhang et al. [65] investigated the use of lake sediment, cinder, and sewage sludge as alternative raw materials in the production of fired bricks. The compressive strength of the manufactured bricks showed significant variation. Brick 1, made entirely from lake sediment, exhibited the highest strength at 33.1 MPa. The compressive strength decreased to 25.4 MPa in Brick 2 with 10% cinder and to 20.5 MPa in Brick 3 with 5% sewage sludge. The decrease in strength is attributed to the higher organic matter and loss on ignition (LOI) for the sludge, which resulted in greater porosity and macro defects during sintering. All brick types met the minimum compressive strength requirement of 20 MPa set by the Chinese Standard (GB/T 5101-2003 [124]), confirming their structural viability for construction use.

On the other hand, Brahim et al. [98] investigated the incorporation of alkali-activated, fly ash-stabilized sediments in CEBs. A significant enhancement of the mechanical performance of CEBs was noticed. Dry compressive strength increased from 2.4 MPa for the unstabilized reference sample to 11.6 MPa at 50% fly ash substitution, while wet compressive strength improved from 0.95 MPa to 6.9 MPa over the same range. This improvement is primarily attributed to the formation of dense aluminosilicate gels that effectively bind sediment particles and reduce porosity. Notably, all stabilized CEBs containing 10% or more fly ash exceeded the 4 MPa thresholds required for load-bearing masonry applications, as defined by the XP P13-901 standard.

Similarly, Larbi et al. [110] demonstrated that the mechanical performance of the CEBs was significantly improved when dredged sediments with glass powder activated with sodium hydroxide were integrated. Among the tested formulations, 40 g of glass powder dissolved in 100 mL of 4 mol NaOH produced the highest compressive strength of 6.63 MPa. This value is above the required limit of CEBs (1.0–2.8 MPa), which makes them suitable for structural purposes. When 30 g of glass was used under the activation conditions, the resulting strengths were between 2.31 and 5.95 MPa, with an average of 4 MPa. Best performance was found at 4% glass content.

Belayali et al. [97] also investigated the potential reuse of dredged sediments from northern France as a primary aggregate for geopolymer-stabilized CEB production. The authors conceived formulations prepared from sediment, fly ash (FA), and ground blast furnace slag (GBFS) activated by 8 M NaOH solution. Based on their findings, the CEBs’ compressive strength increases with a higher fly ash and GBFS ratio. A mix containing 54% cement, 36% FA and 10% GBFS showed a 28-day strength of 16.53 MPa, which is above the minimum required as per specifications. GBFS enhanced strength by contributing additional calcium ions, facilitating the formation of calcium-alumino-silicate hydrate (CASH) and C-S-H gel. These gels reinforced the matrix and improved compaction, confirming the mechanical advantages of geopolymer binders over traditional stabilizers.

Additionally, Serbah et al. [96] examined the reuse of dredged sediments after being mixed with 30% sand. The results showed a significant improvement with both increased compaction energy and reduced moisture. Initially, when compacted at Modified Proctor energy, the unconfined compressive strength (UCS) was 0.7 MPa, which did not meet the minimum standard requirement of 1.6 MPa (NF XP P13-901). However, by increasing the compaction to 25 blows, the UCS increased to 1.8 MPa, which meets the standard. Further testing demonstrated an inverse correlation between water content and compressive strength: as the water percentage decreased from 13.6% (optimum) to 1.95%, the UCS increased sharply from 1.8 MPa to 5.7 MPa. This improvement is attributed to higher suction during drying, which enhances capillary forces between particles and strengthens inter-particle bonds. These findings highlight the critical role of compaction energy and moisture control in optimizing the mechanical performance of compressed earth blocks.

According to Bhairappanavar et al. [57], the compressive strength of dredged material–cement bricks (DM-CBs) depends mainly on the cement content and the compaction pressure. All bricks that have been compacted with 2 MPa or 4 MPa at 10% or 12% cement content passed ASTM C67 at 28 days, with end strength ranging from 11.3 MPa to 16.0 MPa. But the uncompacted specimens failed to reach the minimum requirement of 10.3 MPa. Increased compaction pressure enhances brick strength notably; for example, at 8% cement, strength improved by 41% when compaction pressure increased from 2 MPa to 4 MPa. Similarly, increasing cement content from 8% to 12% led to a 41% strength gain at 4 MPa. These results were attributed to greater density and enhanced formation of hydration products such as calcium silicate hydrate.

As can be noticed, the compressive strength reported among the reviewed studies demonstrates that the mechanical behavior of sediment-based bricks cannot be linked solely to the sediment incorporation ratio, but rather to the combined influence of sediment characteristics and manufacturing conditions. Sediments rich in silica and aluminosilicate minerals generally promote sintering, vitrification, and the formation of a denser ceramic matrix, whereas high organic matter and carbonate contents increase gas evolution during firing, resulting in higher porosity and lower compressive strength. Likewise, particle-size distribution plays a crucial role, as finer sediments improve particle packing and densification, while coarser particles may generate structural discontinuities. The manufacturing process further governs the final mechanical performance. For fired bricks, firing temperature controls mineral transformations and matrix densification, whereas for compressed earth blocks, the type and dosage of stabilizers, compaction pressure, curing conditions, and moisture content become the dominant parameters controlling strength development. Therefore, the wide range of compressive strengths reported in the literature primarily reflects the intrinsic heterogeneity of dredged sediments with differences in processing conditions.

### 4.3. On Thermal Properties

The integration of dredged sediments into construction materials has shown promising results not only in mechanical and environmental performance but also in thermal behavior, an increasingly critical criterion in energy-efficient building design. Globally, buildings account for one-third of total energy consumption, with half of this energy lost through the building envelope, particularly walls [125]. In this context, the thermal conductivity of bricks and earth blocks becomes a key parameter in optimizing energy use, improving indoor comfort, and complying with low-energy building regulations. The ability of these materials to provide effective thermal insulation depends on several factors, including the nature of the constituent materials [126], moisture content [127], and porosity [128].

Several studies have evaluated the impact of sediment incorporation on the thermal properties of bricks (see Table 7). Slimanou et al. [63] investigated fired bricks produced with harbor dredged sediments and demonstrated low thermal conductivity values, ranging from 0.43 to 0.24 W/m·K at 950 °C and from 0.46 to 0.21 W/m·K at 850 °C. These values are within the limits anticipated for insulating materials, ensuring these bricks are thermally appropriate for applications involving heat retention or energy conservation.

**Figure 17:**
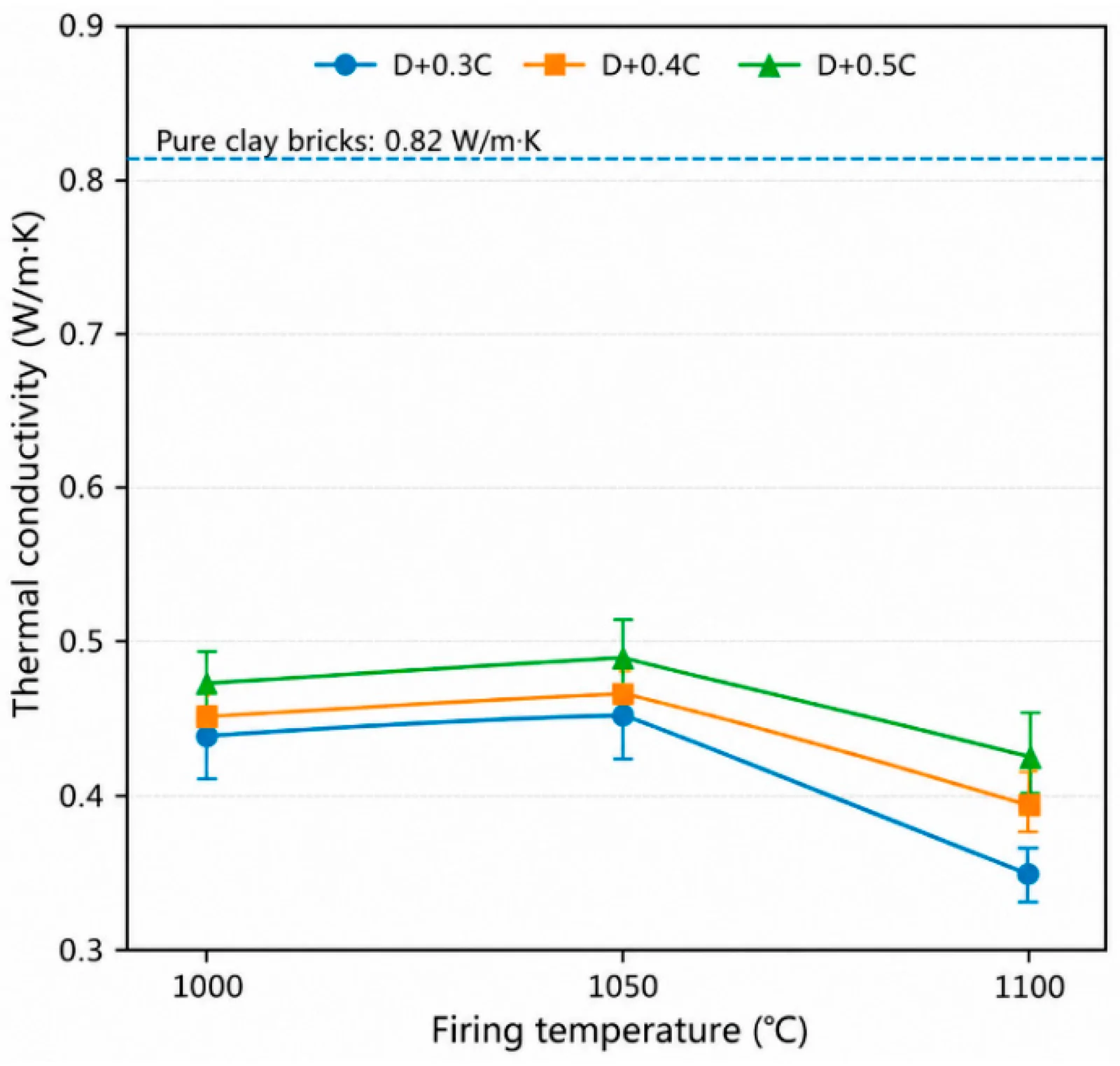
Effect of firing temperature and HDS/clay proportion on the thermal conductivity of fired bricks, reconstructed from Xu et al. [66].

Similarly, Xu et al. [66] evaluated the insulation capacity of fired bricks made from urban river sediments (up to 50% replacement) and reported a reduction of at least 40% in thermal conductivity compared to the conventional bricks, as can be seen in Figure 17. The measured values were within the range of 0.35 to 0.48 W/m·K. This improvement was due to the presence of sediment, along with the enhanced porosity and decreased bulk density. An increase in thermal conductivity was observed as the firing temperatures were increased from 1000 °C to 1050 °C due to matrix densification as well as structural transformations. These findings highlight the importance of optimizing the firing temperature to balance strength and insulation performance.

In addition, Manni et al. [108] studied the thermal behavior of bricks with 0, 10, 30 and 50 percent dredged sediment replacing natural clay. The thermal conductivity of all the samples was found to range from 0.45 to 0.50 W/m·K. This implies that thermal performance remains unaffected after the addition of sediment. Minor variations in thermal diffusivity (0.42–0.55 mm^2^/s) and specific heat capacity (1.06–0.82 J/kg·K) were reported. The higher variability in the mixtures with high sediment content is likely attributed to the intrinsic heterogeneity of sediment.

According to a report by Zhang et al. [65], lake sediment entirely made of brick recorded the highest thermal conductivity of 0.623 W/(m·K). When 10% cinder was added, a reduction to 0.610 W/(m·K) was recorded, while the addition of 5% sewage sludge (0.533 W/(m·K)) reflected a 14% reduction. The results were correlated with increasing porosity and the presence of organic matter from the sludge.

In addition to fired bricks, the use of dredged sediments in compressed earth blocks (CEBs) has also given good thermal results. Brahim et al. [98] studied stabilized sediment-based CEBs using alkali-activated fly ash. As the fly ash content increased, the thermal conductivity and specific heat increased marginally yet remained below conventional values. Therefore, these materials have the potential to be used in low-energy buildings. Moreover, thermal regulation specifications, such as France’s RT2012, mandate a maximum annual energy requirement of 50 kWh/m^2^ (for heating, cooling, lighting and ventilation). The results obtained correlate with higher bulk density and reduced porosity, due to the formation of alkali-activated gels that densify the internal microstructure.

These studies show that dredged sediments can be successfully incorporated into fired bricks and compressed earth blocks while providing thermal properties suitable for energy-efficient building applications. However, the reported thermal performance varies considerably depending on the characteristics of the sediment and the manufacturing conditions. Sediments containing higher organic matter generally promote pore formation during firing, thereby reducing thermal conductivity and improving insulation performance. Conversely, increased densification resulting from higher firing temperatures, lower porosity, or the formation of vitrified phases tends to increase thermal conductivity while simultaneously enhancing mechanical strength. The mineralogical composition and particle-size distribution of the sediments further influence heat transfer by controlling the development of the pore network and the compactness of the ceramic matrix. In the case of compressed earth blocks, the incorporation of stabilizers such as fly ash and alkali activators modifies the microstructure through the formation of cementitious gels, leading to changes in density and thermal conductivity.

### 4.4. Durability Test: Freeze-Thaw

Freeze-thaw durability is a key performance criterion for construction materials exposed to cold and temperate climates. It evaluates the ability of a material to resist deterioration caused by repeated freezing and thawing cycles, during which water retained within the pore network freezes, expands, and generates internal stresses that progressively damage the microstructure. Multiple studies have shown that pore size and pore size distribution of clay bricks are crucially important for their freeze–thaw degradation resistance. Whenever water-saturated bricks are subjected to freezing conditions, pressure develops in the pore network due to ice formation. The pressure can increase as water penetrates the material during thawing periods, which may ultimately produce microcracking, spalling, or surface scaling in subsequent cycles. Table 8 summarizes the weight loss results of previous studies on fired bricks.

Lafhaj et al. [59] performed standardized freeze–thaw tests on clay bricks containing various blends of treated river sediments. Each sample was subjected to 25 cycles of 4-h freezing at −17.8 °C and 15 h thawing in water at 12 °C. Following the cycles, specimens were exposed to air for 24 h and oven-dried again at 105 °C to measure mass loss as an indicator of freeze–thaw damage. No clear correlation was observed between the proportion of treated sediment and the percentage of mass loss. All substitution levels resulted in weight losses of less than 1%, which is below the maximum threshold defined by the French standard [119]. Thus, this confirms a reasonable freeze–thaw resistance regardless of the sediment content or the sediment incorporation ratio. Furthermore, no visible cracking or surface deterioration was reported, confirming that the incorporation of treated sediments did not adversely affect the long-term frost durability of the fired bricks.

Samara et al. [58], who replaced 15% of quartz sand with treated sediments, reported similar findings. All samples exhibited weight losses under 1%, with no signs of cracking or breakage, thereby passing the French freeze–thaw qualification for bricks. The absence of visible surface damage indicates that freeze–thaw resistance should not be assessed solely through mass loss, but also by evaluating surface deterioration after cyclic exposure.

Mezencevova et al. [56] tested bricks composed of 100% dredged sediment and a 50/50 mix with brick clay. Bricks composed of 100% sediment exhibited mass losses of 0.33–0.45%, greater than the 0.16–0.25% mass loss of blended bricks but remained within the ASTM C62 maximum limit of 0.5% for SW grade. No visible deterioration or cracking appeared, confirming their suitability for freeze–thaw conditions. These observations demonstrate that satisfactory freeze–thaw performance can be achieved even at high sediment incorporation levels, provided that the resulting microstructure limits water penetration and internal frost damage.

Bhairappanavar et al. [57] further investigated the freeze–thaw durability of DM-CBs over 50 cycles. Results improved significantly with increased cement content and compaction. The best-performing sample (C12P4) containing 12% cement compressed with a compaction pressure of 4 MPa lost only 0.27% of its weight without any visible damage. Even sample C8P0, which was the least optimized, had a direct drying shrinkage of 0.53%, indicating there was still no failure of the structure. However, approached the ASTM C62 limit. The authors attributed this improvement to the refinement of the pore structure produced by increased cement stabilization and compaction, which reduced capillary water absorption and permeability, thereby limiting frost-induced deterioration. These findings also suggest that freeze–thaw resistance should be interpreted together with residual compressive strength and water absorption rather than mass loss alone.

**Figure 18:**
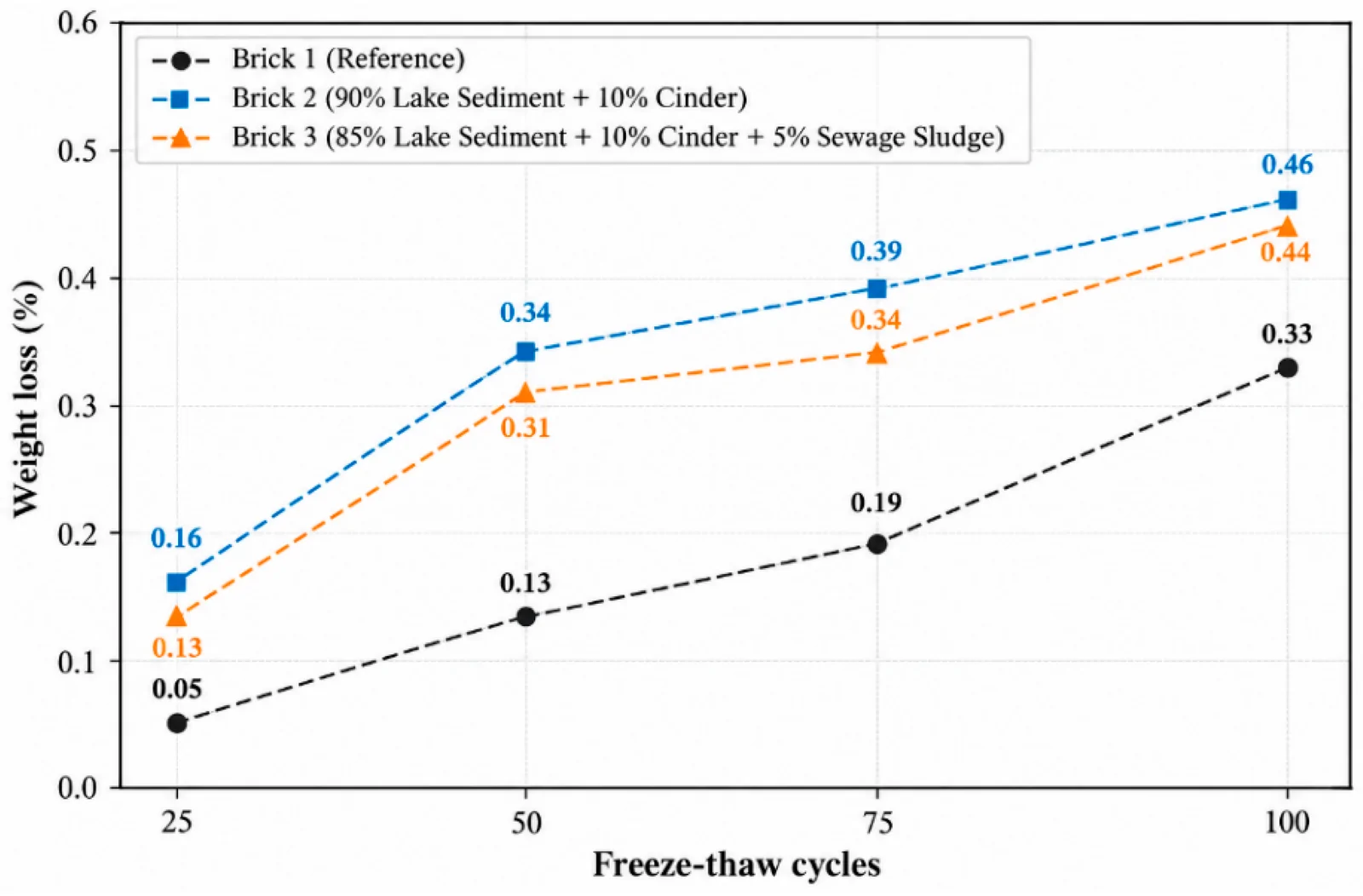
Evolution of weight loss with freeze–thaw cycles for the reference brick and bricks incorporating lake sediment, cinder, and sewage sludge, reconstructed from Zhang et al. [65].

Zhang et al. [65] evaluated the freeze-thaw resistance of the fired bricks after 100 freeze-thaw cycles. All brick types showed values of weight loss significantly below the 2% threshold specified by the Chinese standard GB/T 2542-2012 [129], indicating satisfactory resistance (Figure 18). Brick 1 (100% lake sediment) has the best freeze-thaw performance, presumably attributed to the lower total porosity of Brick 1 and the lower percentage of micro-pores smaller than 2 µm, which reduces the internal hydraulic pressure generated during freezing. In terms of better resistance, Brick 3, having 5% sewage sludge, outperformed Brick 2 with sediment and 10% cinder. Brick 2, having more fine pores, led to a greater moisture content, thus creating internal stress and causing a strength reduction. In contrast, the larger pore size and partial sintering owing to sludge combustion in Brick 3 will allow drainage of water and relieve stress, which favor frost durability. Using scanning electron microscopy (SEM) and mercury intrusion porosimetry (MIP), the authors further demonstrated that pore size distribution and pore connectivity govern freeze–thaw performance more significantly than total porosity alone. Bricks containing a higher proportion of interconnected micropores retained more water and were therefore more susceptible to microcrack development during freeze–thaw cycling, whereas larger and less connected pores acted as pressure-relief reservoirs that reduced frost-induced damage. These observations highlight that the evolution of the pore network during repeated freeze–thaw cycles plays a decisive role in frost deterioration. The progressive increase in pore connectivity facilitates water ingress, promotes hydraulic pressure during ice formation, and accelerates microcrack propagation, ultimately leading to a gradual reduction in the mechanical integrity of the bricks.

The reviewed studies demonstrate that the freeze–thaw durability of sediment-based bricks is governed mainly by the pore structure rather than by the sediment incorporation ratio alone. Although the chemical and mineralogical compositions of dredged sediments influence the final microstructure, the durability is primarily governed by the distribution, connectivity, and size of the pores generated during manufacturing. Sediments with high organic matter or carbonate contents may increase porosity through gas release during firing; however, when appropriate firing conditions promote sufficient vitrification and matrix densification, the resulting pore network remains resistant to freeze–thaw damage. Similarly, for compressed earth blocks, the incorporation of stabilizers together with higher compaction pressures reduces capillary porosity and water ingress, thereby enhancing frost resistance.

Based on the available literature, freeze–thaw durability should preferably be assessed using a multidimensional evaluation framework combining mass loss, residual mechanical strength, pore structure evolution, and surface deterioration. However, no standardized classification system specifically dedicated to sediment-based bricks is currently available, highlighting the need for future research to establish harmonized durability assessment criteria.

### 4.5. Environmental Performance

Bricks manufactured with treated dredged sediments demonstrated high environmental performance, particularly regarding heavy metal immobilization and leaching safety. Many studies, including Lafhaj et al. [59], Samara et al. [58], and Xu et al. [66], have confirmed that leaching tests performed according to the French standards (AFNOR XP X31-210 [130]), the U.S. EPA’s Toxicity Characteristic Leaching Procedure (TCLP), and Chinese standard GB5085.3-2007 [131] show that concentrations of hazardous metals such as cadmium, lead, zinc, copper, and nickel remain well below regulatory thresholds for inert or non-hazardous waste classification.

Lafhaj et al. [59] observed that even at substitution rates up to 45%, zinc concentrations in TCLP leachates did not exceed 4.92 mg/kg, far below the 25 mg/kg threshold. Samara et al. [58] reported similarly favorable results, confirming that the Novosol^®^ treatment process (combining phosphatation and calcination) and subsequent high-temperature firing effectively stabilized contaminants by incorporating them into the matrix, likely through the formation of low-solubility oxides or vitrified phases. In addition to chemical stability, surface durability was also verified. None of the tested bricks showed signs of efflorescence following standardized moisture exposure, due to the inclusion of 7 g/kg of barium carbonate, which successfully converted soluble salts into insoluble ones. The sediment-blended bricks exhibited higher pH levels after firing, further reducing metal solubility in potential leaching scenarios.

Xu et al. [66] confirmed these findings, showing that bricks containing up to 70% urban river sediment exhibited no detectable arsenic, cadmium, or mercury and had total concentrations of lead and zinc below 1 mg/L, with higher firing temperatures (e.g., 1050 °C) enhancing immobilization.

Moreover, Ducman et al. [109] tested bricks with heavy metal leachates at concentrations well below the limits stipulated in European Council Decision 2003/33/EC, indicating that they would be safe for the environment at substitution levels of up to 50%.

Beyond leaching safety, the environmental benefits of sediment-based bricks have also been evaluated through Life Cycle Assessment (LCA). Although the number of LCA studies specifically addressing dredged sediment bricks remains limited, the available investigations demonstrate that incorporating dredged sediments can reduce the environmental footprint of brick production. However, this reduction depends on the manufacturing process, pretreatment requirements, and the system boundaries considered in the assessment.

**Figure 19:**
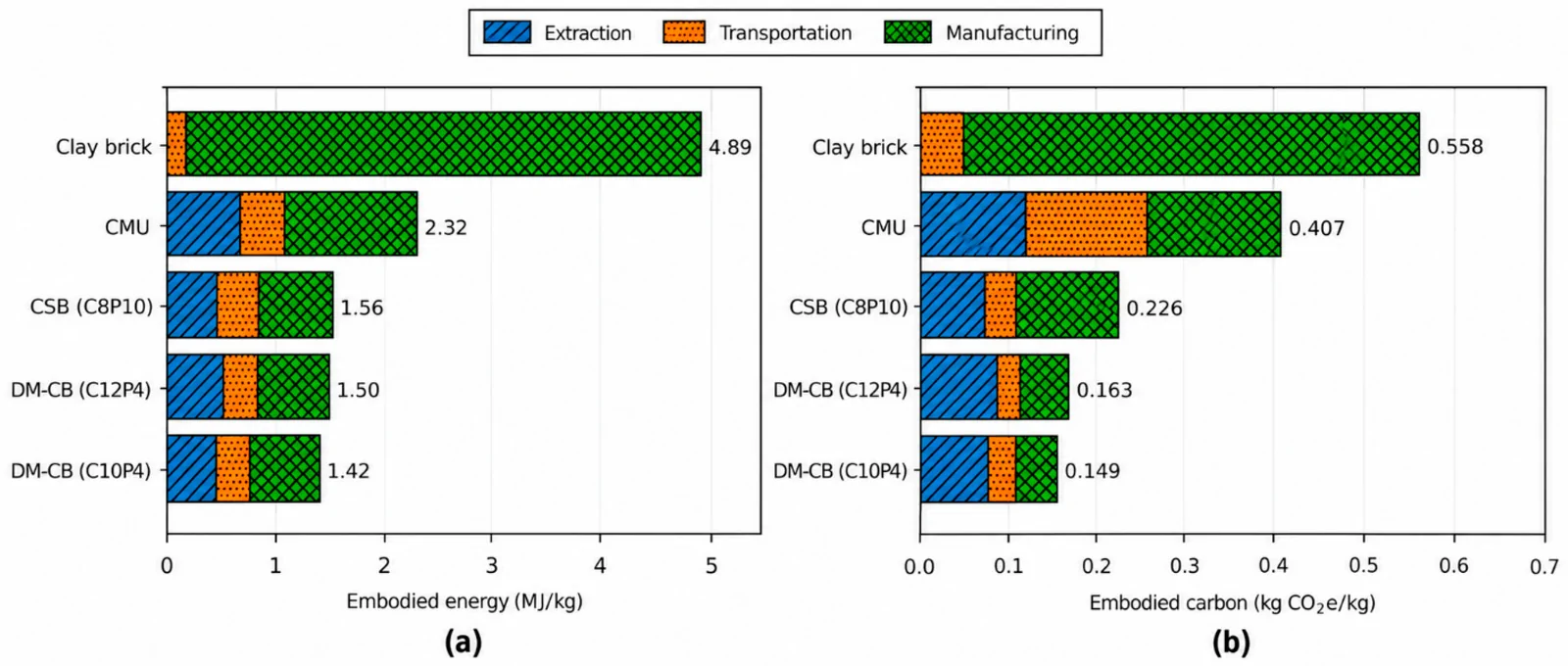
Embodied energy (**a**) and embodied carbon (**b**) of conventional and dredged-material-based bricks from raw-material extraction, transportation, and manufacturing, reconstructed from data reported by Bhairappanavar et al. [57].

Research has shown that dredged material–cement bricks (DM–CBs) have a lower environmental impact than masonry units. Bhairappanavar et al. [57] reported that the embodied energy varied between 1.41 and 1.49 MJ/kg, much less than that of fired clay brick, which equals 4.8 MJ/kg as shown in Figure 19a. Additionally, the carbon emissions were reduced to 0.15–0.16 kg CO_2_ /kg (see Figure 19b). These improvements result from the dual advantage of reusing dredged material, a waste product, and minimizing or eliminating high-temperature processes.

One can notice the environmental potential of unfired sediment-based bricks, where energy savings are primarily attained by eliminating the energy-intensive firing stage. Similarly, Ong et al. [132] performed a cradle-to-gate LCA on compressed bricks manufactured with reservoir sediments and reported lower environmental impacts than conventional compressed clay bricks across the assessed impact categories, further confirming the environmental advantages of unfired sediment-based masonry units.

Moreover, the LCA analysis conducted by Manni et al. [108] showed a clear environmental advantage in partially substituting natural clay (NC) with dredged sediments (DS) in brick manufacturing. Using the ReCiPe 2016 Midpoint Hierarchy method, a cradle-to-gate assessment revealed that incorporating up to 50% DS as a replacement for NC resulted in a 2.8% reduction in the global warming (GW) score. Even more significant were the reductions in mineral resource scarcity (−49.6%), human non-carcinogenic toxicity (−29.1%), and terrestrial ecotoxicity (−36.7%). These improvements are related to the preservation of raw clay and the reuse of dredged waste. The environmental impact decreased consistently with higher DS content across nearly all 18 assessed impact categories, validating the feasibility of DS-enhanced bricks as a lower-impact alternative to conventional clay bricks.

Nevertheless, the reduction in global warming potential remained relatively modest as the high-temperature firing process dominates the overall energy demand and greenhouse gas emissions associated with brick manufacturing. Comparable observations were reported by Božič et al. [133], whose cradle-to-gate LCA of industrial fired clay bricks incorporating Drava River sediments demonstrated that replacing virgin clay with dredged sediments improved the overall environmental performance while maintaining technical compliance. However, the firing stage remained the principal contributor to the total environmental impacts.

The comparison between these studies demonstrates that the environmental benefits of dredged sediment valorization cannot be generalized. Unfired bricks generally exhibit greater reductions in embodied energy and carbon emissions as they eliminate the firing stage, whereas fired bricks still require significant energy for drying and sintering. Furthermore, the overall environmental performance is strongly influenced by both the intrinsic characteristics of the dredged sediments and the selected manufacturing process. Environmental safety depends on the initial contamination level, chemical and mineralogical composition, and the effectiveness of pretreatment techniques in immobilizing hazardous elements before or during firing.

Likewise, the overall environmental benefits identified by life-cycle assessment are closely related to the sediment incorporation ratio, transportation distance, pretreatment requirements, and energy consumption during manufacturing. While replacing natural clay with dredged sediments generally reduces raw material extraction and waste disposal, these benefits may be partially offset when extensive dewatering, drying, decontamination, or thermal pretreatments are required. Therefore, the environmental performance of sediment-based bricks should be evaluated through case-specific life-cycle assessments considering both contaminant immobilization and the complete production chain, rather than being assumed solely from the replacement of natural raw materials by dredged sediments. The optimal valorization strategy consequently depends on the specific physicochemical characteristics of each sediment source and the selected manufacturing route.

Similarly, the economic viability of sediment-based bricks is expected to depend on many of the same factors, including sediment moisture content, transportation distance, pretreatment requirements, sediment incorporation ratio, and local energy prices. As only a limited number of studies have reported comprehensive techno-economic assessments, the cost competitiveness of sediment-based bricks cannot yet be generalized and should be evaluated on a case-by-case basis.

This conclusion is consistent with the broader sustainability perspective proposed by Sala et al. [134], who emphasized that CO_2_ emission reduction strategies should be assessed within an integrated life-cycle framework considering environmental, economic, and energy-related aspects. Consequently, comprehensive life cycle and techno-economic assessments remain essential for identifying the most sustainable dredged sediment valorization strategies under specific local conditions.

## 5. Challenges and Recommendations for Brick Construction

### 5.1. Challenges in Utilizing Dredged Sediments in Brick Production

As can be noticed from the previous section on the different studies conducted, the incorporation of dredged sediments into brick manufacturing presents both environmental and technical complexities. The main challenge is the heterogeneous nature of dredged sediments. Changes in origin result in variations in the particle size distribution, mineralogical composition, and moisture content, which complicate their valorization in brick formulations.

Another critical challenge is the pollutant levels in sediments that require pre- and post-environmental risk assessment. Many studies have shown that contaminants could be stabilized by firing or geopolymerization processes, the use of untreated sediments may release leachable contaminants or compromise the mechanical performance of the bricks.

From a technical standpoint, mechanical limitations can arise when using sediments with poor cohesion or weak pozzolanic activity. In the absence of suitable binders or stabilizers, bricks containing sediments display reduced compressive strength and limited durability under environmental conditions. This is specifically undesirable for compressed earth blocks (CEBs) as strength is an essential requirement of load-bearing walls.

Moreover, the lack of public awareness and regulatory acceptance are significant barriers to sediment reuse. Although it has environmental benefits and technical feasibility, sediment is often classified as waste, which delays its acceptance among stakeholders and end-users. Regulatory standards for sediment-based bricks are still being developed, which creates uncertainty for manufacturers and limits commercialization opportunities.

Finally, sediment management, which incorporates dredging, transportation, treatment and incorporation into manufacturing, requires careful planning and resource coordination to meet logistical and infrastructural needs. In regions where dredging locations are distant from production facilities, sediment reuse will not be cost-effective, posing further constraints for large-scale implementation.

### 5.2. Strategic Recommendations for Sustainable Implementation

Despite all challenges discussed earlier, the reuse of dredged sediments in brick construction has significant potential if suitable technical support and policy measures are initiated. A key step towards this approach is the development of a standardized protocol for dredged sediment treatment. This includes mechanical sieving, dewatering, and chemical screening. Such procedures ensure consistency and reliability in brick quality. Establishing regional characterization databases, similar to the aggregate classification system used in concrete, can evaluate sediment suitability for specific applications.

In addition, incorporating an alkali-activated binder, especially from industrial waste fly ash (FA) or ground blast furnace slag (GBFS), has demonstrated considerable promise. Through the employment of these geopolymers, the compressive strength and durability of bricks improve, as well as their carbon footprint reduces. This indicates the need for continued research into geopolymer formulations adapted to sediment characteristics.

Policy support is also vital. Governments and local authorities should consider the reclassification of dredged sediments as an alternative raw material rather than waste when used under controlled conditions. Regulatory frameworks like green procurement policies, R&D grants and tax credits can encourage industrial adoption. Public-private partnerships and pilot-scale projects can also be useful for technology validation.

Finally, public awareness and education campaigns are important for curing social uncertainty. Encouraging the benefits to the environment, including reduced CO_2_ emissions, landfill diversion, and conserving natural clay. Pilot projects, especially in public infrastructure, can serve as proof-of-concept for larger initiatives.

## 6. Conclusions

The reuse of dredged sediments in the construction sector represents a promising solution for addressing the environmental, economic, and regulatory challenges caused by sediment disposal, while simultaneously contributing to the development of sustainable building materials. This review has comprehensively summarized the current knowledge and practices regarding the valorization of dredged sediments in brick manufacturing.

Dredged sediments possess properties that can be suitable for the formulation of construction materials. The mineralogical composition, which is rich in clay, silt and siliceous materials, aligns well with the requirements of traditional raw materials used in brick manufacturing. However, due to the variability of their composition according to sediment origin, no single formulation is universally applicable. Instead, the selection of an appropriate valorization route should be based on appropriate physicochemical, mineralogical, and environmental characterization. When sediments are properly treated, they can replace partially or entirely natural clays, thus reducing virgin material extraction, and contribute to the circular economy of the construction sector.

Nevertheless, there are significant challenges associated with valorizing dredged sediments in construction applications. The source of the sediment plays a major role in determining its characteristics. The most critical aspect is their intrinsic heterogeneity, as differences in grain size, organic matter content, contaminant levels, and moisture content require systematic characterization and quality control before industrial reuse can be considered.

The presence of contaminants, particularly heavy metals and organic pollutants, creates additional barriers to sediment reuse. The suitability of each sediment for construction applications still needs to be evaluated, although thermal processes such as firing in brick kilns or sintering already have the potential to immobilize many contaminants. Regulatory ambiguities surrounding the classification of sediments as waste or secondary raw materials continue to delay broader acceptance and integration into mainstream markets.

From a technical perspective, the mechanical and physical properties of sediment-based bricks are strongly influenced by sediment composition and processing conditions. In many studies, compressive strength values ranged from 2 to 15 MPa depending on the binder used, sediment treatment, and curing method. Thermal conductivity and water absorption behavior also varied, with high porosity often leading to lower thermal conductivity but reduced mechanical strength and durability. Stabilization with cement, lime, or alkali-activated binders has shown considerable potential in improving these properties while maintaining environmental benefits.

Moreover, the durability of sediment-based construction materials has been demonstrated in freeze-thaw cycles, water immersion tests, and leaching experiments. However, the long-term performance remains dependent on the sediment source, formulation, and manufacturing process. These evaluations confirm that, when properly formulated, such materials meet or exceed standard performance requirements. The potential to produce low-carbon bricks, especially with geopolymer technologies and industrial by-products, further enhances the environmental application of sediment reuse.

The effective large-scale implementation of sediment-based bricks requires the adoption of standardized pretreatment and quality-control procedures to reduce the variability of raw sediments. Depending on the sediment characteristics, pretreatment operations such as dewatering, drying, sieving, washing, grinding, thermal treatment, contaminant stabilization, or blending with conventional clay may ensure consistent processing behavior and product quality. In addition, optimizing sediment incorporation ratios, firing conditions, and stabilization strategies according to the characteristics of each sediment source represent a practical approach for improving the reproducibility, durability, and industrial applicability of sediment-based bricks. The development of harmonized characterization protocols and engineering guidelines would therefore facilitate technology transfer from laboratory research to full-scale industrial production.

It is also important to distinguish between the two principal valorization pathways identified in this review. Fired sediment bricks generally provide higher mechanical strength, lower water absorption, and greater durability owing to the sintering process, making them particularly suitable for structural and exterior applications exposed to demanding service conditions. In contrast, non-fired compressed earth blocks (CEBs) offer significant environmental advantages by eliminating the energy-intensive firing stage, thereby reducing embodied energy and associated CO_2_ emissions. When appropriately stabilized, CEBs represent an attractive solution for low-rise construction, non-load-bearing walls, and sustainable earthen buildings. Therefore, the selection between fired and non-fired sediment-based products should be guided by the intended application, required performance, local environmental conditions, and sustainability objectives.

Considering global sustainability goals and the urgent need for environmentally responsible construction practices, dredged sediments can represent a viable alternative to conventional raw materials for brick production. Their successful integration into construction applications, especially bricks and compressed earth blocks, demands a coordinated effort that includes standardized protocols for treatment and characterization, supportive regulatory frameworks, and market-driven innovation.

The findings of the reviewed studies demonstrate that dredged sediments should be considered a valuable secondary resource rather than waste requiring disposal, provided that they are appropriately characterized, treated, and managed according to their physicochemical and environmental characteristics. With continued research, stakeholder engagement, standardized engineering practices, and supportive regulatory frameworks, sediment valorization has the potential to contribute to more sustainable construction by reducing landfill disposal, conserving natural resources, and lowering the carbon footprint of the building sector.

## Figures and Tables

**Table 1:** Natural formation and sedimentation of sediments [13].

From Land Areas	From Marine Areas
▪ Erosion of the riverbed, bank erosion, and repositioning of sediment within waterways [46].	▪ Rocks and soil particles that have been washed away from lands and the debris of marine organisms, submarine volcanic deposits, compounds that precipitated from seawater, and extraterrestrial matter [47].
▪ Weathering and erosion of minerals, organic matter, and soil from upstream regions and riverbanks [48].	▪ A diverse mixture of materials deposited on the seafloor, originating from continental erosion, volcanic activity, biological productivity, hydrothermal vents, and cosmic debris [49].
▪ Mud, sand, and silt accumulated in navigable channels, bay inlets, and marinas as a result of upstream sediment erosion [50].	▪ Deposits collected on seafloor, comprising debris from land-based erosion, biological remains, organic matter, minerals accumulated from seawater, and volcanic materials [51].
▪ Soil erosion from highlands and hillslopes, episodic events like mass wasting and erosion in fire-affected areas, and stream-bank erosion in the stream-corridor [52].	
▪ Sheet, rill, and gully erosion from highland areas, along with ravine, bluff, and stream-bank erosion [53].	

**Table 2:** Comparative chemical and mineralogical compositions of representative dredged sediments and conventional raw materials.

Reference	Raw Material	Source	SiO_2_	Al_2_O_3_	Fe_2_O_3_	CaO	MgO	Na_2_O	K_2_O	LOI	Main Mineral Phases
Mezencevova et al. (2012) [56]	Ceramic clay	Georgiabrick plant, USA	60.45	20.06	8.13	0.07	0.98	0.35	2.63	6.06	Quartz, kaolinite
Bhairappanavar et al. (2021) [57]	Clayey soil (shale)	Ohio, USA	58.29	18.92	5.86	0.28	1.67	0.76	4.10	7.35	Clay minerals
Samara et al. (2009) [58]	Quartz sand	Northern France	≈100	–	–	–	–	–	–	–	Quartz
Mezencevova et al. (2012) [56]	River dredged sediment	Savannah Harbor, USA	54.79	17.23	7.40	2.03	1.62	0.61	1.36	12.81	Quartz, kaolinite, feldspars
Lafhaj et al. (2008) [59]	River dredged sediment (treated)	Dampremy–Charleroi Canal, Belgium	54.33	8.22	14.27	10.89	1.72	0.68	1.49	3.68	Quartz, hematite, calcite, anorthite
Snellings et al. (2016) [16]	Marine dredged sediment	Port of Antwerp, Belgium	37.0	10.0	9.0	12.0	1.6	0.5	2.0	22.00	Quartz, illite/montmorillonite, kaolinite, amorphous phase
Ozer-Erdogan et al. (2016) [60]	Marine dredged sediment	Ambarlı Port, Türkiye	61.9	15.1	3.6	9.6	2.5	1.7	2.6	–	Quartz, illite/montmorillonite
Amar et al. (2021) [42]	Marine dredged sediment	Grand Port Maritime de Dunkerque, France	51.9	8.2	9.3	22.1	2.0	2.0	1.9	9.90	Quartz, illite/montmorillonite, kaolinite
El Mahdi Safhi et al. (2019) [61]	Marine dredged sediment	Grand Port Maritime de Dunkerque, France	38.8	11.0	13.3	23.3	2.7	2.1	2.1	5.90	Quartz, calcite, illite, kaolinite
Hamer and Karius (2002) [62]	Marine dredged sediment	Bremen Harbor, Germany	63.0	11.0	8.3	1.8	0.9	1.9	1.6	10.00	Quartz, feldspars, illite, kaolinite
Slimanou et al. (2020) [63]	Harbor dredged sediment	Bejaia Port, Algeria	46.8	14.0	5.3	17.2	2.6	1.2	2.2	10.40	Quartz, calcite, illite, kaolinite
Aouad et al. (2012) [64]	River dredged sediment	Scarpe Canal, France	55.5	6.5	3.3	5.8	0.6	0.6	1.8	10.20	Quartz, calcite, illite, kaolinite
Zhang et al. [65]	Lake dredged sediment	Changdang Lake, China	69.74	16.20	5.78	1.29	2.11	1.24	2.13	5.11	Quartz
Xu et al. (2014) [66]	River dredged sediment	Qinhuai River, China	55.3	12.3	4.9	5.4	2.2	1.1	2.0	13.30	Quartz, feldspars, illite, kaolinite
Lim et al. (2019) [67]	Harbor dredged sediment	Kaohsiung Harbor, Taiwan	57.8	18.7	7.7	2.0	2.6	2.1	3.9	6.60	Quartz, albite, illite, chlorite
He et al. (2020) [68]	River dredged sediment	Xunsi River, China	62.6	19.5	6.5	3.2	2.0	0.9	–	–	Quartz, kaolinite, illite
Junakova et al. (2017) [69]	Reservoir sediment	Klusov Reservoir, Slovakia	68.6	18.0	5.2	1.1	2.4	–	3.0	7.20	Quartz, albite, muscovite, chlorite
Mymrin et al. (2017) [30]	Marine dredged sediment	Paranaguá Port, Brazil	71.0	10.1	3.8	2.6	1.1	4.5	3.4	3.60	Quartz, feldspars, mica, clay minerals

**Table 3:** Granulometric distribution of dredged sediments from different locations.

Reference	Sediment’s Origin	Sand (%) (>63 µm)	Silt (%) (<63 µm)	Clay (<2 µm) (%)
Dorleon et al. (2024) [18]	10 harbors, South of France	31	58	
Boutouil (1998) [73]	Le Havre port, Normandie Region, France	4.80–7.08	83.61–84.90	9.31–10.30
Marot (1997) [74]	Estuary of the Loire and the Seine, France	10	90	
Miraoui et al. (2012) [75]	Dunkerque Port, North of France	0.15–2.1	90.2–90.24	7.65–8.52
Zhao et al. 2018 [76]		4	74	22
Agostini et al. (2007) [48]		36	59	5
Benzerzour et al. (2017) [17]		0	58	42
El Mahdi Safhi et al. (2020) [77]		10	70	20
Rozière et al. (2015) [78]	Dampremery port-Chareloi, Belgium	65	34	1
Doni et al. (2018) [79]	Livorno port, Italy	63	37	
Ferrans et al. (2021) [80]	Malmfärden port, Sweden	10–20	80–90	
Bel Hadj Ali et al. (2014) [81]	Rades port, Tunisia	20	80	

**Table 4:** Past studies on fired bricks and blocks with different types of sediments.

Reference	Sediment Origin	Treatment Before Incorporation in Bricks	Content (%wt)	Bricks Type	Shaping Technique	Experimental Scale	Year
Samara et al. [58]	River sediments, Dampremy-Charleroi, Belgium	Novosol^®^ process (phosphatation + calcination)	15% (quartz sand replacement)	Fired bricks (1000 °C)	Extrusion	Industrial	2009
Serbah et al. [96]	Bakhadda Dam, Algeria	Granulometry improved by adding 30% sand	70% sediment + 30% sand	Compressed Earth blocks (CEBs)	Pressing	Laboratory	2018
Mezencevova et al. [56]	Savannah Harbor, USA	None	50–100%	Fired bricks (900–1000 °C)	Extrusion	Laboratory	2012
Xu et al. [66]	Qinhuai River, China	Sieved to <2 mm	50–70%	Insulating fired bricks (1000–1100 °C)	Pressing	Laboratory	2014
Lafhaj et al. [59]	North France rivers	Novosol^®^ (phosphatation + calcination)	0–45%	Fired clay bricks (1010 °C)	Extrusion	Laboratory	2008
Hamer and Karius [62]	Harbour Basin of Bremen, Germany	None	50%	Fired bricks (1050 °C)	Pressing	Industrial	2002
Chiang et al. [104]	Wushe Reservoir, Taiwan	None	80–100%	Fired bricks (1000–1150 °C)	Pressing	Laboratory	2008
Romero et al. [105]	Astilleros, Cuchia and Rasos, Spain	None	100%	Fired (900–1200 °C)	Pressing	Laboratory	2008
Slimanou et al. [63]	Rmilia Harbor, Algeria	Dried and sieved (125 μm)	0–100%	Fired clay bricks (850–950 °C)	Pressing	Laboratory	2020
Brahim et al. [98]	MEL, Northern France	Sieved to 0–5 mm	50–100%	Stabilized CEBs (fly ash + NaOH)	Pressing	Laboratory	2022
Hussain et al. [106]	Harbor Dunkirk port, France	Mixing Dunkirk sediments with hemp shiv	90–95% + 0–5% fiber	Adobe bricks	Pressing	Laboratory	2022
	River, Usumacinta, Mexico	Mixing sediments with palm oil flower fibers					
El Fgaier et al. [107]	North France riversNorth France rivers	Novosol^®^ process	15% (quartz and sand replacement)	Fired clay bricks (1010 °C)	Extrusion	Industrial	2013
Zhang et al. [65]	Changdang Lake, China	None	100%	Fired bricks (950 °C)	Extrusion	Laboratory	2016
			90% (+10% cinder)				
			80% (+10% cinder and 5% sewage sludge)				
Manni et al. [108]	Canal Port of Ravenna, Italy	Sieving and washing	0–50%	Fired clay bricks (1000 °C)	Extrusion	Laboratory	2021
Bhairappanavar et al. [57]	Cuyahoga River, USA	None	88–92%	DM-Cement Bricks (DM-CBs)	Pressing	Laboratory	2021
Ducman et al. [109]	Drava River, Slovenia	None	0–50%	Fired clay bricks (950 °C)	Extrusion	Laboratory	2022
Larbi et al. [110]	Oran and Sidi Lakhdar, Algeria	None	70% Oran port + 30% Sidi Lakhdar port	Compressed Earth blocks (CEBs)	Pressing	Laboratory	2022
Belayali et al. [97]	Aire-sur-la-Lys, North France	Sieving (5 mm)	54–90%	Compressed Earth blocks (CEBs)	Pressing	Laboratory	2022
Belmonte et al. [35]	Glaciogene marine clay, Ilulissat, Greenland	Addition of 10% chamotte	Marine clay with 10% chamotte	Fired red bricks (1030–1080 °C)	Extrusion	Pilot scale	2024

Experimental scale: Laboratory = specimens manufactured and tested under controlled laboratory conditions; Pilot = production using pilot or semi-industrial manufacturing facilities; Industrial = production carried out in commercial manufacturing plants under industrial operating conditions.

**Table 5:** Physical properties of bricks blended with dredged sediments.

Reference	Formulated Bricks	Water Absorption (%)	Porosity (%)	Firing Shrinkage (%)	Bulk Density (Kg/m^3^)	Loss on Ignition (%)	Year
Hamer and Karius [62]	Fired bricks with 50% sediments	11–15	-	-	1720–1800	-	2002
Chiang et al. [104]	Fired bricks with 80–100% sediments	0–24	0–39	9–32	1800–2450	-	2008
Romero et al. [105]	Fired bricks with 100% sediments	8–30	10–20	0–5	1950–2050	-	2008
Mezencevova et al. [56]	Fired bricks with 50–100% sediments	7.9–18.8	-	10.1–15.7	-	8.8–11.2	2012
Xu et al. [66]	Fired bricks with 50–70% sediments	17–27.5	-	0.53–10.62	1230–1450	9.13–11.73	2014
Serbah et al. [96]	CEBs with 70% sediments	-	-	14	1910	-	2018
Lafhaj et al. [59]	Fired bricks with 0–45% sediments	5.3–10.39	-	-	-	-	2008
Slimanou et al. [63]	Fired bricks with 0–100% sediments	14.37–21.45	-	0.19–1.09	1764–1854	-	2020
Samara et al. [58]	Fired bricks with 15% sediments	7.5	15.4	10	-	-	2009
Brahim et al. [98]	CEBs with 50–100% sediments	-	28–38	-	1600–1850	-	2022
El Fgaier et al. [107]	Fired bricks with 15% sediments	8.51	17	7	-	-	2013
Zhang et al. [65]	Fired bricks with 80–100% sediments	15.4–18.7	-	5.35–6.45	1633–1710	5.3–11.6	2016
Bhairappanavar et al. [57]	Fired bricks with 88–92% sediments	13.73–17.92	-	-	-	-	2021
Ducman et al. [109]	Fired bricks with 0–50% sediments	17.2–25.1	-	0–1.3	1520–1760	-	2022
Larbi et al. [110]	CEBs with 100% sediments	16.2–21.42	-	-	2620–2750	-	2022

**Table 6:** Compressive strengths of bricks incorporating dredged sediments.

Reference	Formulated Bricks	Compressive Strengths (MPa)	Year
Hamer and Karius [62]	Fired bricks with 50% sediments	18–24	2002
Chiang et al. [104]	Fired bricks with 80–100% sediments	29.5–117	2008
Romero et al. [105]	Fired bricks with 100% sediments	5–34	2008
Mezencevova et al. [56]	Fired bricks with 50–100% sediments	8.3–29.4	2012
Xu et al. [66]	Fired bricks with 50–70% sediments	2–12.56	2014
Lafhaj et al. [59]	Fired bricks with 0–45% sediments	25.58–43.70	2008
Slimanou et al. [63]	Fired bricks with 0–100% sediments	71.96–127.7	2020
Samara et al. [58]	Fired bricks with 15% sediments	22–36	2009
Brahim et al. [98]	CEBs with 50–100% sediments	2.4–11.6	2020
Serbah et al. [96]	CEBs with 70% sediments	1.8–5.7	2018
El Fgaier et al. [107]	Fired bricks with 15% sediments	36	2013
Zhang et al. [65]	Fired bricks with 80–100% sediments	20.5–33.1	2016
Bhairappanavar et al. [57]	Fired bricks with 88–92% sediments	7.99–16	2021
Ducman et al. [109]	Fired bricks with 0–50% sediments	25–33	2022
Larbi et al. [110]	CEBs with 100% sediments	1.01–6.63	2022
Belayali et al. [97]	CEBs with 54–90% sediments	4.56–16.53	2022

**Table 7:** Thermal properties of bricks integrating dredged sediments.

Reference	Formulated Bricks	Thermal Conductivity (W/(m∙K))	Specific Heat Capacity (J/(kg∙K))	Year
Xu et al. [66]	Fired bricks with 50–70% sediments	0.35–0.48	-	2014
Slimanou et al. [63]	Fired bricks with 0–100% sediments	0.24–0.43	-	2020
Brahim et al. [98]	CEBs with 50–100% sediments	0.38–0.58	1010.56–1120.23	2022
El Fgaier et al. [107]	Fired bricks with 15% sediments	0.167	-	2013
Zhang et al. [65]	Fired bricks with 80–100% sediments	0.533–0.623	-	2016
Manni et al. [108]	Fired bricks with 0–50% sediments	0.45–0.5	820–1060	2021

**Table 8:** Freeze-thaw results of bricks incorporating dredged sediments.

Reference	Bricks	Freeze-Thaw: Weight Loss (%)	Year
Samara et al. [58]	Fired bricks with 50–100% sediments	After 50 cycles: 0.36	2009
Mezencevova et al. [56]	Fired bricks with 50–100% sediments	After 50 cycles: 0.16–0.45	2012
Lafhaj et al. [59]	Fired bricks with 0–45% sediments	After 25 cycles: 0.17–0.36	2008
Zhang et al. [65]	Fired bricks with 80–100% sediments	After 100 cycles: 0.05–0.46	2016
Bhairappanavar et al. [57]	Fired bricks with 88–92% sediments	After 50 cycles: 0.27–0.53	2021

## Data Availability

No new data were created or analyzed in this study. Data sharing is not applicable to this article.
